# Development and Validation of a Personalized Prognostic Prediction Model for Patients With Spinal Cord Astrocytoma

**DOI:** 10.3389/fmed.2021.802471

**Published:** 2022-01-18

**Authors:** Sheng Yang, Xun Yang, Huiwen Wang, Yuelin Gu, Jingjing Feng, Xianfeng Qin, Chaobo Feng, Yufeng Li, Lijun Liu, Guoxin Fan, Xiang Liao, Shisheng He

**Affiliations:** ^1^Department of Orthopedics, Shanghai Tenth People's Hospital, Tongji University School of Medicine, Shanghai, China; ^2^Spinal Pain Research Institute, Tongji University School of Medicine, Shanghai, China; ^3^Department of Orthopedics, The First Affiliated Hospital, Shenzhen University, Shenzhen, China; ^4^Shenzhen Second People's Hospital, Shenzhen, China; ^5^Shanghai East Hospital, Tongji University School of Medicine, Shanghai, China; ^6^Key Laboratory of Computational Neuroscience and Brain-Inspired Intelligence, Ministry of Education, Shanghai, China; ^7^Institute of Science and Technology for Brain-Inspired Intelligence, Behavioral and Cognitive Neuroscience Center, Fudan University, Shanghai, China; ^8^The First Clinical Medical College of Guangzhou University of Chinese Medicine, Guangzhou, China; ^9^College of Artificial Intelligence, Guangxi University for Nationalities, Nanning, China; ^10^Department of Orthopedics, The Eighth Affiliated Hospital, Sun Yat-sen University, Shenzhen, China; ^11^National Key Clinical Pain Medicine of China, Huazhong University of Science and Technology Union Shenzhen Hospital, Shenzhen, China; ^12^Guangdong Key Laboratory for Biomedical Measurements and Ultrasound Imaging, School of Biomedical Engineering, Shenzhen University Health Science Center, Shenzhen, China; ^13^Department of Pain Medicine, Shenzhen Municipal Key Laboratory for Pain Medicine, The 6th Affiliated Hospital of Shenzhen University Health Science Center, Shenzhen, China

**Keywords:** spinal tumor, astrocytoma, prognostic factor, survival prediction, nomogram, SEER

## Abstract

**Background:**

The study aimed to investigate the prognostic factors of spinal cord astrocytoma (SCA) and establish a nomogram prognostic model for the management of patients with SCA.

**Methods:**

Patients diagnosed with SCA between 1975 and 2016 were extracted from the Surveillance, Epidemiology, and End Results (SEER) database and randomly divided into training and testing datasets (7:3). The primary outcomes of this study were overall survival (OS) and cancer-specific survival (CSS). Cox hazard proportional regression model was used to identify the prognostic factors of patients with SCA in the training dataset and feature importance was obtained. Based on the independent prognostic factors, nomograms were established for prognostic prediction. Calibration curves, concordance index (C-index), and time-dependent receiver operating characteristic (ROC) curves were used to evaluate the calibration and discrimination of the nomogram model, while Kaplan-Meier (KM) survival curves and decision curve analyses (DCA) were used to evaluate the clinical utility. Web-based online calculators were further developed to achieve clinical practicability.

**Results:**

A total of 818 patients with SCA were included in this study, with an average age of 30.84 ± 21.97 years and an average follow-up time of 117.57 ± 113.51 months. Cox regression indicated that primary site surgery, age, insurance, histologic type, tumor extension, WHO grade, chemotherapy, and post-operation radiotherapy (PRT) were independent prognostic factors for OS. While primary site surgery, insurance, tumor extension, PRT, histologic type, WHO grade, and chemotherapy were independent prognostic factors for CSS. For OS prediction, the calibration curves in the training and testing dataset illustrated good calibration, with C-indexes of 0.783 and 0.769. The area under the curves (AUCs) of 5-year survival prediction were 0.82 and 0.843, while 10-year survival predictions were 0.849 and 0.881, for training and testing datasets, respectively. Moreover, the DCA demonstrated good clinical net benefit. The prediction performances of nomograms were verified to be superior to that of single indicators, and the prediction performance of nomograms for CSS is also excellent.

**Conclusions:**

Nomograms for patients with SCA prognosis prediction demonstrated good calibration, discrimination, and clinical utility. This result might benefit clinical decision-making and patient management for SCA. Before further use, more extensive external validation is required for the established web-based online calculators.

## Introduction

Primary spinal cord tumor is rare in patients, and for this reason, the relevant statistics are still lacking (1). It was reported that the incidence of primary spinal cord tumor (PSCT) was 10–15 times less than that of primary intracranial tumors, which accounts for ~5–15% of all spinal tumors and 2–4% of all primary tumors of the central nervous systems (CNS) (2, 3). According to the anatomical location, PSCT can be classified as extradural, intradural extramedullary, and intramedullary (4). Intramedullary spinal cord tumor (IMSCT) (8–10% of all PSCT) includes three most common types including ependymomas (60–70%), astrocytoma (30–40%), and hemangioblastoma (3–8%) (2, 4). According to Tobin et al. (5), astrocytoma is one of the most frequent malignancies that is frequently seen in the intramedullary tumor. Compared with the other two tumor types, astrocytoma carries a worse prognosis. Nakamura et al. (6) reported that the 5-year survival rate for spinal cord astrocytoma (SCA) was 68% while 36% for 10-year survival. Clinic symptoms of astrocytoma occur with increased intracranial pressure, resulting from mass effect or hydrocephalus (7, 8). In adult patients, astrocytoma can show the atypical location and early recurrence, presenting aggressive behaviors (8–10). However, it usually lead to a better prognosis than children with astrocytoma (11).

The clinical treatment options for SCA, especially for high-grade SCA, are very limited, and the prognosis is difficult to predict (12). Treatment options for astrocytoma include gross total resection (GTR), subtotal resection (STR), and radiation therapy (13–15). However, GTR cannot often be reached due to the infiltrative nature of the tumor and the lack of a clear dissection plane (5). Babu et al. (16) reported that 37% of patients presented worse neurological outcomes after surgical resection and 54.8% of patients even emerged new neurological deficits. In order to assist the patient management, Tabash (17) predicted child patients' survival and reported the incidence rates of pilocytic astrocytoma in the spinal cord, but there is still a lack of research on the prognosis of SCA in adults. Zou et al. (18) conducted a multi-institutional cohort study to investigate the prognosis and treatment of SCA in both children and adults, but they did not develop the prognosis prediction for SCA.

There were limited studies for the survival prediction of spinal cord tumors (17, 19, 20). Diaz-Aguilar et al. pointed out that studies on the prediction of spinal astrocytoma were particularly rare (21). Most of the previous studies contained a small sample size and used single-center studies, which provide poor guidance for clinical prognosis (22–26). Therefore, the aim of this study was to establish a systematic and effective prognostication model that meets the needs of personalized prediction in clinical medicine, based on a large sample size data set from the Surveillance, Epidemiology, and End Results (SEER) Program of the National Cancer Institute.

## Materials and Methods

### Source of Databases

The SEER registry was used to search the incidence and survival information of all registered cases with malignant astrocytoma. Maintained by National Cancer Institute, SEER collects data including age, race, diagnosis years, primary tumor site, histology, grade, distant metastasis, and treatment regimens, and survival months from 18 population-based cancer registries and reflects cancer statistics of 28% of the population of the whole United States. Ethical approval is not required for this study because the SEER database is free of any sensitive patient information or identifiers.

### Eligibility Criteria

Astrocytoma-related data between 1975 and 2016 was collected based on the International Classification of Disease for Oncology Version 3 (ICD-O-3) coding system. We only extracted malignant astrocytoma patients whose tumor primary site is in the spinal cord (C72.0) and excluded those in spinal meninges (C70.1) or cauda equina (C72.1). Patients diagnosed with glioblastoma (histology codes 9440/3, 9441/3) were also excluded due to the relatively large survival rate difference between glioblastomas and non-glioblastoma astrocytoma (27). Other exclusion criteria are as follows: (1) Patients were diagnosed as Pleomorphic xanthoastrocytoma (histology codes 9424/3). (2) Patients were diagnosed without histological confirmation. (3) Patients were recorded with the wrong WHO grade. (4) Patients those had autopsy or death certificates only. (5) Patients with SEER cause-specific death classification as “missing/unknown COD” or “N/A not a first tumor.” Finally, the concrete type of histology, including cases are astrocytoma, NOS (9400/3), anaplastic astrocytoma (9401/3), protoplasmic astrocytoma (9410/3), gemistocytic astrocytoma (9411/3), fibrillary astrocytoma (9420/3), and pilocytic astrocytoma, malignant (9421/3).

### Clinical Information

Patients' clinical information in this study included age, gender, race, Hispanic, insurance, marital status, residence, histologic type, WHO grade, tumor size, tumor extension, primary site surgery, post-operation radiotherapy (PRT), and chemotherapy. Socioeconomic information such as “at least bachelor's degree,” “families below poverty,” “unemployed,” “median household income,” and “cost of living index” were also extracted based on the US Census 2013–2017 American Community Survey 5-year data files, which were collected at the county level. Overall survival (OS) and cancer-specific survival (CSS) were used to measure survival outcomes.

### Nomogram Development and Validation

Data were randomly divided into training and testing datasets (7:3). For the training dataset, univariable and multivariable Cox regression analyses were applied to estimate the independent prognostic variables for OS and CSS, which were indicated by the hazard ratio (HR) and corresponding 95% CI. Then the identified independent variables were ranked to output the relative importance in the final model. Each variable's contribution was measured as the partial chi-square statistic minus the variable degrees of freedom (χ^2^ – df). Visual nomograms predicting 5-/10-year OS and CSS were conducted based on the final model for OS and CSS, respectively. The testing dataset was then used as external validation for the performance of the nomogram. The calibration curve was used to evaluate the calibration, while the overall concordance index (C-index) and time-dependent receiver operating characteristic (ROC) curve were used for discrimination. The clinical utility of the nomogram was evaluated using decision curve analysis (DCA). Performance of nomogram was also compared with single indicators like age, histologic type, and WHO grade, among others. Then, X-tile software was utilized to divide patients into high-risk, medium-risk, and low-risk groups according to the nomogram's calculated total points. Kaplan-Meier (KM) survival curves and log-rank tests were used to discriminate the different risk groups of OS and CSS.

### Statistical Analysis

This study followed the Strengthening the Reporting of Observational Studies in Epidemiology (STROBE) reporting guideline and the Transparent Reporting of a multivariable prediction model for Individual Prognosis or Diagnosis (TRIPOD) statement. Continuous variables were expressed as mean (SD) and compared using one-way ANOVA, while categorical variables were expressed as count (percentage) and compared using the Chi-square test. Multiple imputations were used to estimate the missing values (100 imputations). The SEER^*^Stat (version 8.3.9; https://seer.cancer.gov/data/) software was used for data extraction. Then, X-tiles program (version 3.5; developed by Yale University) was used to identify the best cut-points for the numerical variables. All other analyses were carried out by R software (version 3.6.1; R Foundation for Statistical Computing, Vienna, Austria; https://www.r-project.org). Two-tailed *p* < 0.05 were considered statistically significant.

## Results

### Patient Characteristics

**Figure 1:**
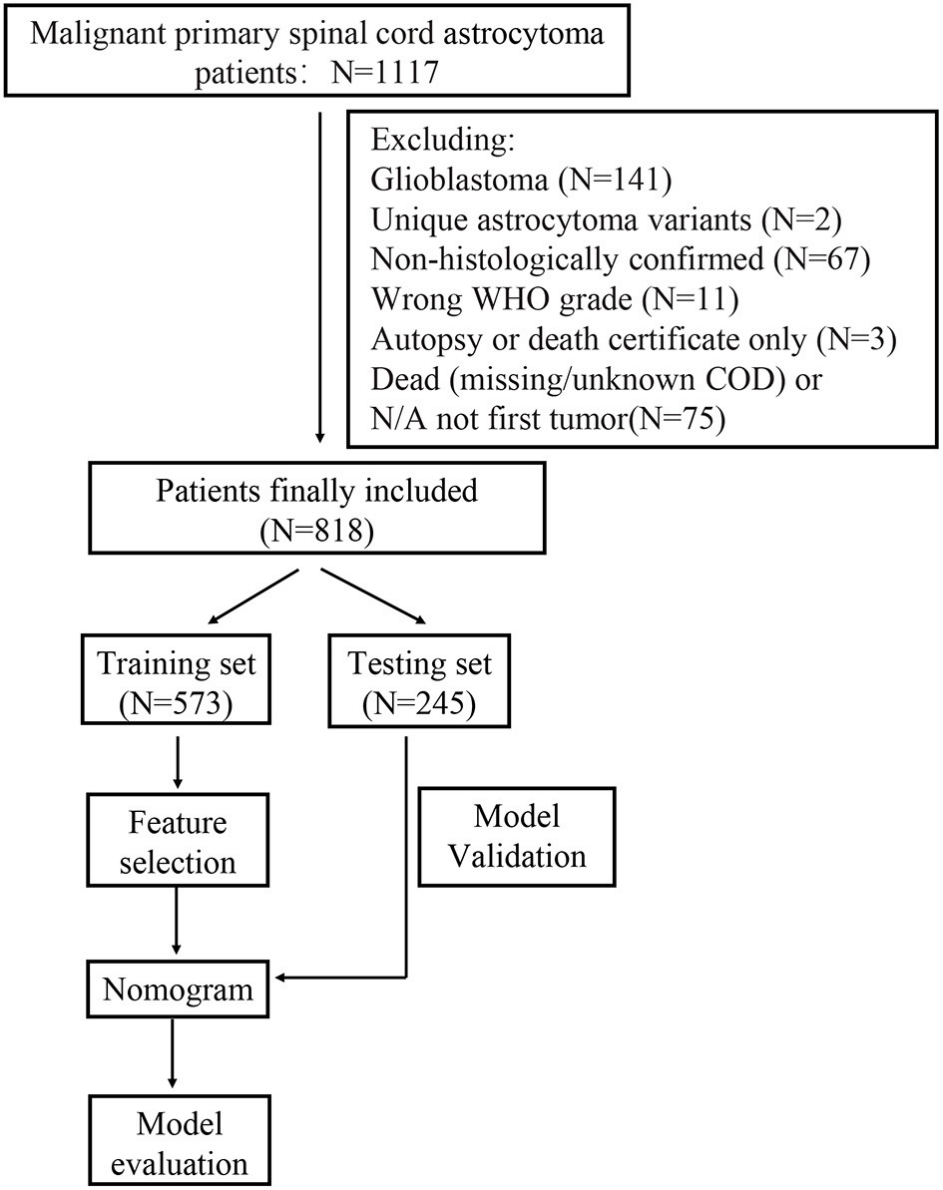
Workflow of the patient selection and model development.

We finally identified a total of 818 patients diagnosed with SCA between 1975 and 2016 from the SEER. The workflow of patient selection was delineated in Figure 1. Demographically, 478 patients (58.4%) were alive at the last follow-up time for OS (336 for the training dataset and 142 for the testing dataset) while 569 patients (69.6%) for CSS (398 for the training dataset and 171 for the testing dataset). The survival months were 117.57 ± 113.51. The average age of patients with SCA was 30.84 ± 21.97, 478 (58.4%) were male and 314 (38.4%) were married. Histologically, the majority of patients (*N* = 404, 49.4%) were diagnosed with astrocytoma, not otherwise specified (NOS), and 263 (32.2%) were diagnosed with pilocytic astrocytoma. Moreover, 312 (38.1%) patients were diagnosed with grade I according to the WHO grading system, and 328 (40.1%) and 178 (21.8%) were diagnosed with grade II and III, respectively. Tumor extension of patients were localized (*N* = 731, 89.4%), regional (*N* = 56, 6.8%), and distant (*N* = 31, 3.8%). Other baseline characteristics of patients were presented in Table 1. Statistical tests between the baseline characteristics of training and testing data set indicated no significant difference (*P* > 0.05).

**Table 1:** Characteristics of patients in training dataset and testing dataset.

**Characteristics**	**Level**	**Whole dataset** **(*N* = 818)**	**Training dataset (*N* = 573)**	**Testing dataset (*N* = 245)**	***P*-value**
Year of diagnosis (%)	1970s	45 (5.5)	35 (6.1)	10 (4.1)	0.942
	1980s	119 (14.5)	87 (15.2)	32 (13.1)	
	1990s	154 (18.8)	110 (19.2)	44 (18.0)	
	2000s	322 (39.4)	218 (38.0)	104 (42.4)	
	2010s	178 (21.8)	123 (21.5)	55 (22.4)	
OS (%)	Alive	478 (58.4)	336 (58.6)	142 (58.0)	0.984
	Dead	340 (41.6)	237 (41.4)	103 (42.0)	
CSS (%)	Alive	569 (69.6)	398 (69.5)	171 (69.8)	0.995
	Dead	249 (30.4)	175 (30.5)	74 (30.2)	
Survival months [mean (SD)]		117.57 (113.51)	121.27 (116.40)	108.90 (106.18)	0.361
Age [mean (SD)]		30.84 (21.97)	30.86 (21.50)	30.80 (23.08)	0.999
Gender (%)	Female	340 (41.6)	236 (41.2)	104 (42.4)	0.945
	Male	478 (58.4)	337 (58.8)	141 (57.6)	
Race (%)	American Indian/Alaska Native	5 (0.6)	4 (0.7)	1 (0.4)	0.363
	Asian or Pacific Islander	55 (6.7)	41 (7.2)	14 (5.7)	
	Black	111 (13.6)	88 (15.4)	23 (9.4)	
	White	647 (79.1)	440 (76.8)	207 (84.5)	
Hispanic (%)	No	718 (87.8)	505 (88.1)	213 (86.9)	0.892
	Yes	100 (12.2)	68 (11.9)	32 (13.1)	
Insurance (%)	Insured	492 (60.1)	342 (59.7)	150 (61.2)	0.919
	Uninsured/Medicaid	326 (39.9)	231 (40.3)	95 (38.8)	
Marital status (%)	Married	314 (38.4)	228 (39.8)	86 (35.1)	0.687
	Separated/divorced/widowed	70 (8.6)	45 (7.9)	25 (10.2)	
	Single/unmarried	434 (53.1)	300 (52.4)	134 (54.7)	
Residence (%)	Metropolitan	740 (90.5)	518 (90.4)	222 (90.6)	0.944
	Rural/urban adjacent to metro area	45 (5.5)	30 (5.2)	15 (6.1)	
	Rural/urban not adjacent to metro area	33 (4.0)	25 (4.4)	8 (3.3)	
At least bachelors degree (%) [mean (SD)]		33.05 (10.80)	32.95 (10.75)	33.29 (10.95)	0.92
Families below poverty (%) [mean (SD)]		10.21 (4.45)	10.25 (4.43)	10.13 (4.53)	0.94
Unemployed (%) [mean (SD)]		6.94 (2.14)	6.97 (2.12)	6.86 (2.20)	0.796
Median household income (in thousand) [mean (SD)]		65.91 (16.50)	65.80 (16.53)	66.16 (16.46)	0.96
Cost of living index (in thousand) [mean (SD)]		1.03 (0.16)	1.04 (0.16)	1.03 (0.16)	0.882
Histologic type (%)	Anaplastic astrocytoma	96 (11.7)	59 (10.3)	37 (15.1)	0.213
	Astrocytoma, NOS	404 (49.4)	299 (52.2)	105 (42.9)	
	Diffuse astrocytoma	55 (6.7)	34 (5.9)	21 (8.6)	
	Pilocytic astrocytoma	263 (32.2)	181 (31.6)	82 (33.5)	
WHO grade (%)	I	312 (38.1)	218 (38.0)	94 (38.4)	0.632
	II	328 (40.1)	238 (41.5)	90 (36.7)	
	III	178 (21.8)	117 (20.4)	61 (24.9)	
Tumor size (mm) (%)	<28	419 (51.2)	301 (52.5)	118 (48.2)	0.519
	≥28	399 (48.8)	272 (47.5)	127 (51.8)	
Tumor extension (%)	Distant	31 (3.8)	22 (3.8)	9 (3.7)	0.948
	Localized	731 (89.4)	509 (88.8)	222 (90.6)	
	Regional	56 (6.8)	42 (7.3)	14 (5.7)	
Primary site surgery (%)	Gross total resection	166 (20.3)	117 (20.4)	49 (20.0)	0.925
	No surgery	151 (18.5)	104 (18.2)	47 (19.2)	
	Partial resection	391 (47.8)	269 (46.9)	122 (49.8)	
	Surgery, NOS	110 (13.4)	83 (14.5)	27 (11.0)	
Postoperation radiotherapy (%)	No	540 (66.0)	376 (65.6)	164 (66.9)	0.936
	Yes	278 (34.0)	197 (34.4)	81 (33.1)	
Chemotherapy (%)	No/Unknown	674 (82.4)	473 (82.5)	201 (82.0)	0.985
	Yes	144 (17.6)	100 (17.5)	44 (18.0)	

Baseline characteristics of the dataset before and after imputation were both summarized in Supplementary Table 1 and no significant difference was detected between the two datasets. Missing data were described in Supplementary Table 2. KM curves of training and testing datasets were demonstrated for both OS and CSS in Supplementary Figure 1. No statistically significant difference was detected between the survival of training and testing datasets in the log-rank test (*P* = 0.497 for OS and *P* = 0.862 for CSS).

### Prognostic Variables and Relative Importance

**Figure 2:**
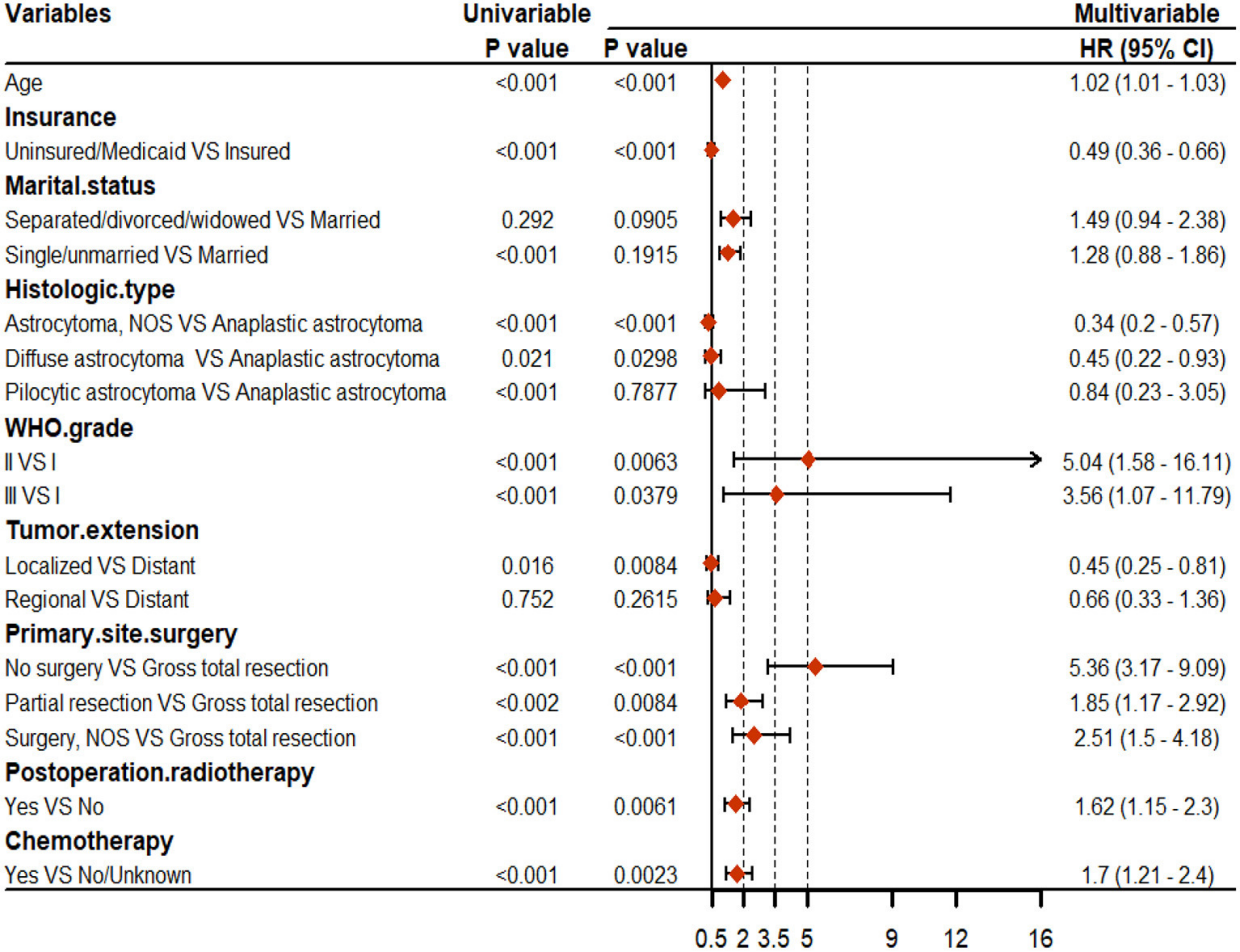
Results of the univariable and multivariable Cox regression analyses for overall survival (OS).

For OS, age, insurance, marital status, histologic type, WHO grade, tumor extension, and primary site surgery, PRT, and chemotherapy were found to be significant factors based on univariable Cox analyses (*P* < 0.05). Results of the multivariable Cox analysis (*P* < 0.05) showed only the age, insurance, histologic type, WHO grade, tumor extension, and primary site surgery, PRT, and chemotherapy to be independent prognostic factors (Figure 2). Primary site surgery was identified to be the most critical risk factor, followed by age, insurance, histologic type, and tumor extension, among others (**Figure 4A**).

**Figure 3:**
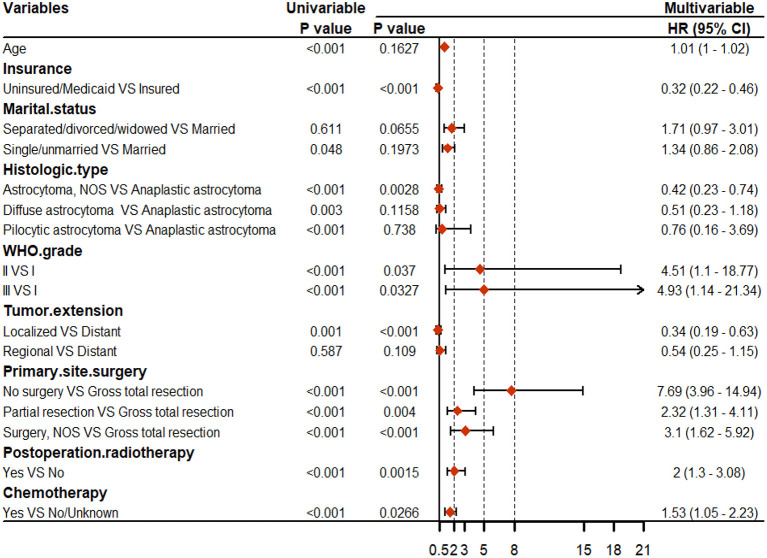
Results of the univariable and multivariable Cox regression analyses for cancer-specific survival (CSS).

**Figure 4:**
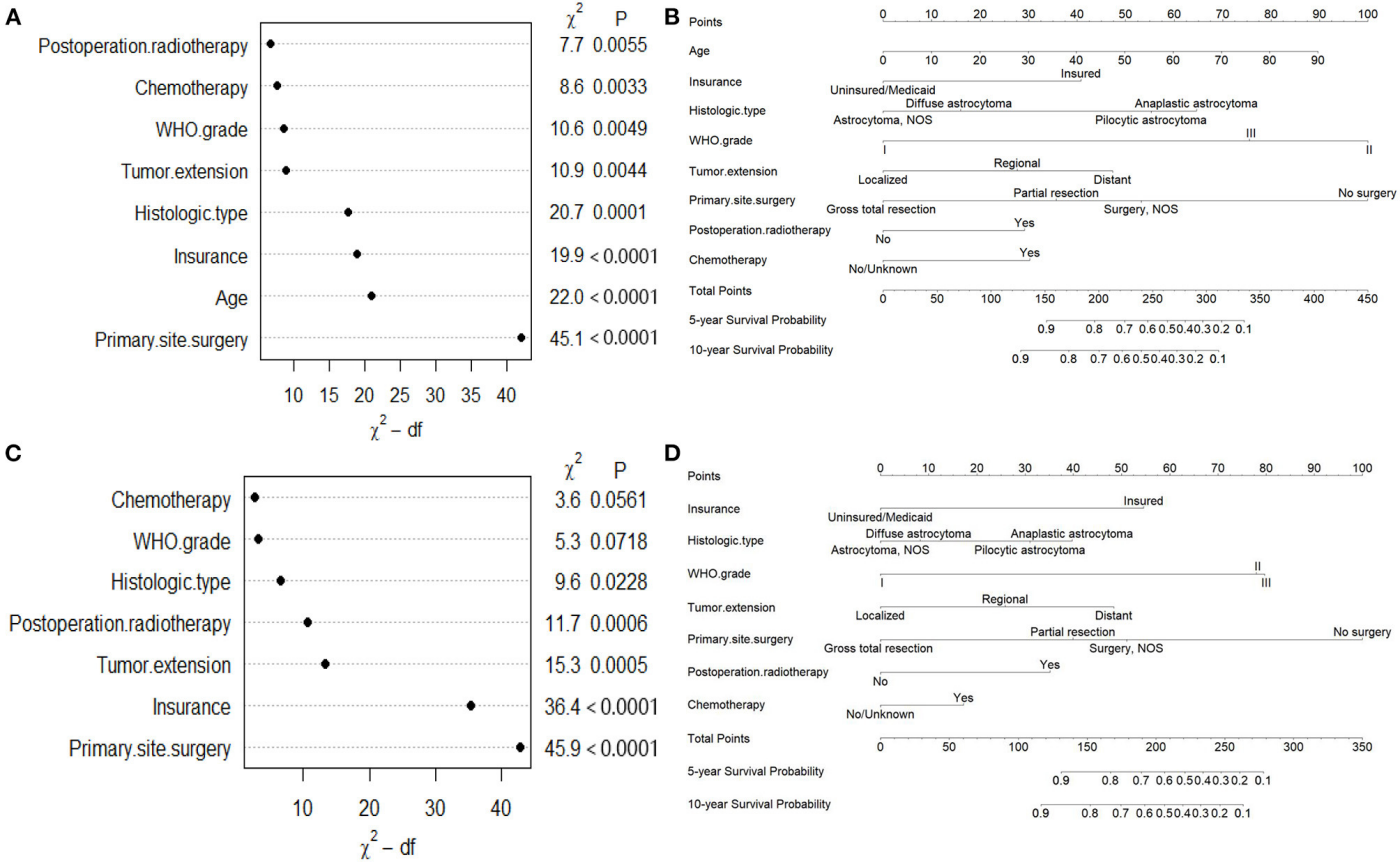
Variable importance and nomograms of **(A,B)** OS and **(C,D)** CSS.

For CSS, age, insurance, marital status, histologic type, WHO grade, tumor extension, and primary site surgery, PRT, and chemotherapy were found to be significant factors based on univariable Cox analyses (*P* < 0.05). Results of the multivariable Cox analysis (*P* < 0.05) showed only the insurance, histologic type, WHO grade, tumor extension, and primary site surgery, PRT, and chemotherapy to be independent prognostic factors (Figure 3). Primary site surgery was identified to be the most critical risk factor, followed by insurance, tumor extension, PRT, histologic type, WHO grade, and chemotherapy (Figure 4C). The complete form of univariable Cox regression analyses for OS and CSS was presented in Supplementary Table 3.

### Nomogram Development and Validation

Based on the independent prognostic variables, nomograms were constructed to predict the probability of the 5- and 10-year OS and CSS, respectively (Figures 4B,D). Score assignment for variables included in the nomograms was shown in Supplementary Table 5.

**Figure 5:**
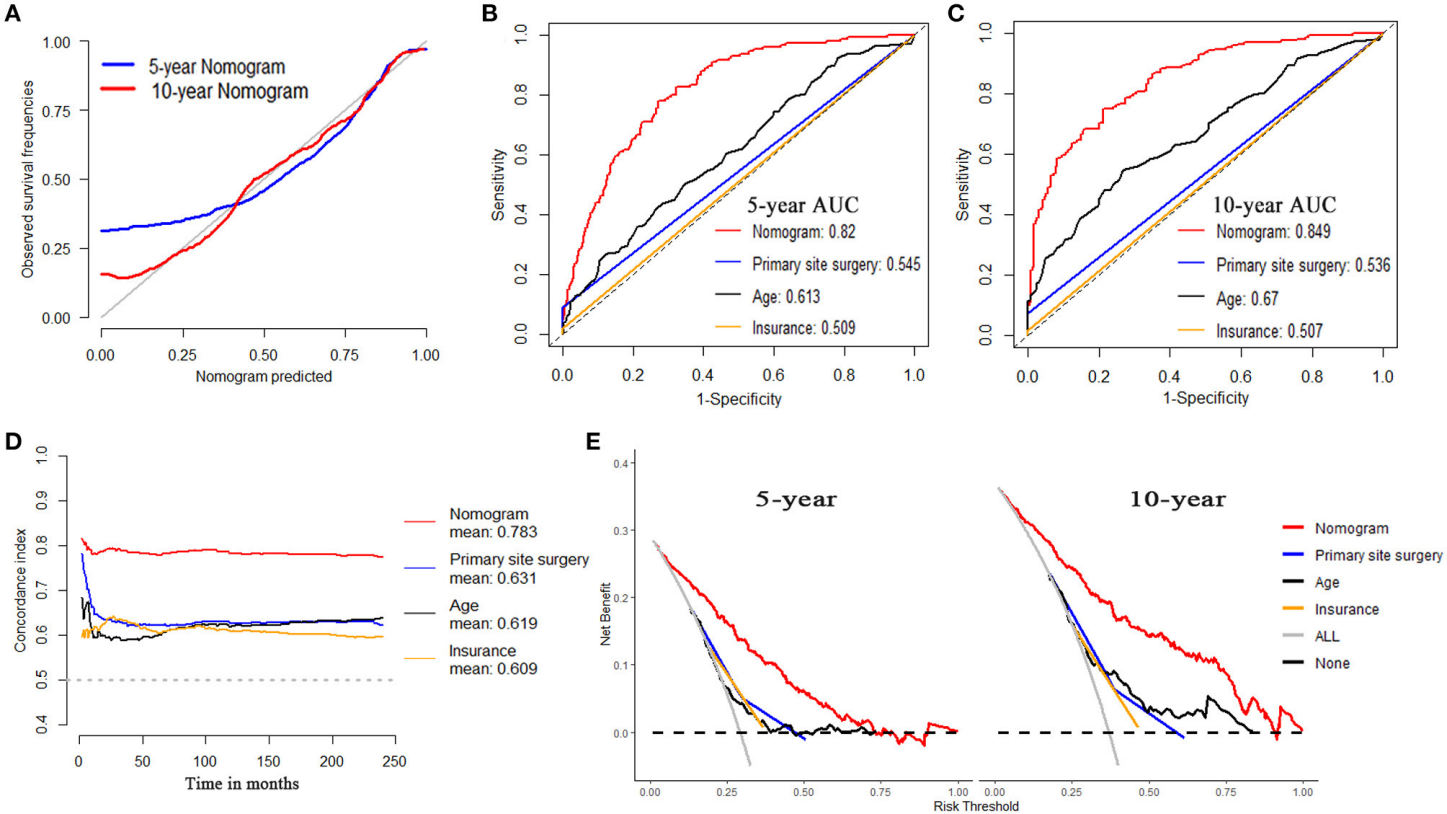
Evaluation of the nomogram on training dataset for OS. **(A)** 5- and 10-year calibration plots of the nomogram. **(B)** 5-year and **(C)** 10-year area under the curve (AUC) for receiver operating characteristic (ROC) curves of Nomogram, Primary site surgery, Age, and Insurance. **(D)** Overall concordance index (c-index) of the nomogram, primary site surgery, age, and insurance. **(E)** 5- and 10-year decision curve analysis (DCA) of the nomogram, primary site surgery, age, and insurance.

**Figure 6:**
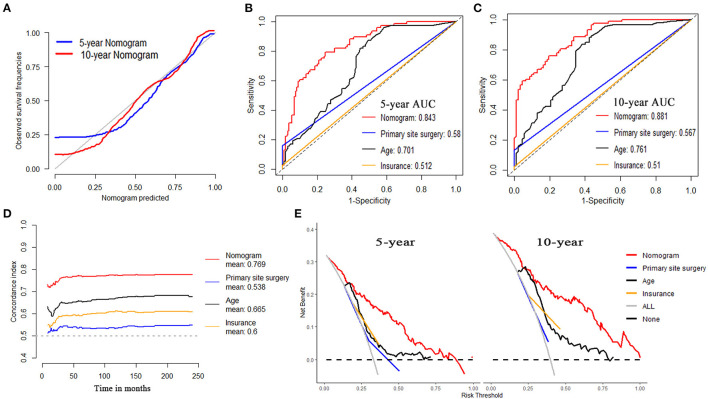
Evaluation of the nomogram on testing dataset for OS. **(A)** 5- and 10-year calibration plots of the nomogram. **(B)** 5-year and **(C)** 10-year AUC for ROC curves of the nomogram, primary site surgery, age, and insurance. **(D)** Overall c-index of the nomogram, primary site surgery, age, and insurance. **(E)** 5- and 10-year DCA of the nomogram, primary site surgery, age, and insurance.

For OS, internal validation was conducted on the training dataset while external validation was on the testing dataset. The nomogram demonstrated good consistency between the observed survival rates and nomogram-predicted results of 5- and 10-year OS, as illustrated in the calibration plot (Figures 5A, 6A). Five- and ten-year area under the curves (AUCs) showed that the nomogram had better performance than single indicators with the 5-year AUCs of 0.82 and 0.843 for training and testing dataset, respectively (Figures 5B, 6B), while 10-year AUCs of 0.849 and 0.881 (Figures 5C, 6C). Overall c-indexes at different follow-up times showed that the nomogram had the best discrimination than other single indicators with the mean c-index of 0.783 and 0.769 for training and testing dataset, respectively (Figures 5D, 6D). Furthermore, DCA curves indicated that the nomogram had the best clinical net benefit than other single indicators in 5- and 10-year OS (Figures 5E, 6E). Further results show that the 5-year area under the decision curves (AUDCs) were 0.087 and 0.114 for the training and testing dataset, respectively, while 10-year AUDCs were 0.155 and 0.184. Complete evaluation results of the nomogram prediction compared to single indicators were presented in Supplementary Table 6.

For CSS, internal validation was also conducted on the training dataset while external validation was on the testing dataset. The nomogram demonstrated good consistency between the observed survival rates and nomogram-predicted results of 5- and 10-year CSS, as illustrated in the calibration plot (Supplementary Figures 2A, 3A). The 5- and 10-year AUCs showed that the nomogram had better performance than single indicators with the 5-year AUCs of 0.851 and 0.834 for training and testing dataset, respectively (Supplementary Figures 2B, 3B), while 10-year AUCs of 0.87 and 0.858 (Supplementary Figures 2C, 3C). Overall c-indexes at different follow-up times showed that the nomogram had the best discrimination than other single indicators with the mean c-index of 0.806 and 0.762 for training and testing dataset, respectively (Supplementary Figures 2D, 3D). Furthermore, DCA curves indicated that the nomogram had the best clinical net benefit than other single indicators in 5- and 10-year CSS (Supplementary Figures 2E, 3E). Five-year AUDCs were 0.089 and 0.084 for training and testing datasets, respectively, while 10-year AUDCs were 0.136 and 0.124. Complete evaluation results of the nomogram prediction compared to single indicators were presented in Supplementary Table 7.

To further evaluation for the recognition ability of nomograms at different follow-up times, we calculated the overall AUCs for OS and CSS on both the training dataset and the testing dataset. Time-dependent curves for AUCs were plotted out in Supplementary Figure 4.

### Risk Discrimination and Online Calculator

**Figure 7:**
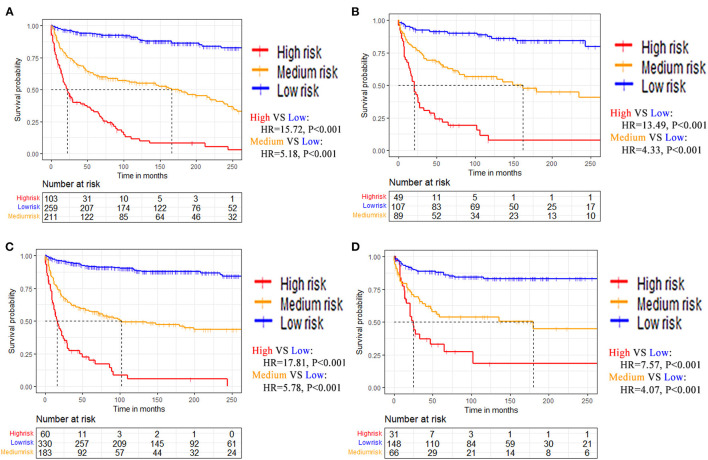
Kaplan-Meier survival curves of patients stratified by risk for **(A)** Training dataset of OS. **(B)** Testing dataset of OS. **(C)** Training dataset of CSS. **(D)** Testing dataset of CSS.

Patients were divided into high-risk, medium-risk, and low-risk groups according to the nomogram calculated total points. The best cutoff points were obtained from patients' total points based on the training dataset with X-tile software (Supplementary Figure 5). KM curves and log-rank tests demonstrated high discrimination of different risk groups (Figure 7).

To achieve the convenience and practicability of clinical management and decision making, we further developed web-based online calculators for prognostic prediction of patients with SCA for OS and CSS, respectively (Supplementary Figure 6). The web tools could be accessed at https://vincent–y.shinyapps.io/Online_nomogram_for_SCA_prediction_OS/ for SCA OS prediction and https://vincent–y.shinyapps.io/Online_nomogram_for_SCA_prediction_CSS/ for CSS prediction. The estimated survival profiles for a hypothetical patient were shown as examples of the web-based calculators in Supplementary Figure 7 (for OS) and Supplementary Figure 8 (for CSS).

## Discussion

Spinal cord astrocytoma (SCA) is a rare intramedullary tumor and there are only limited studies that have been conducted for the survival prediction of patients with SCA. Due to its rarity, clinical risk factors and treatment strategies are still controversial. Most of the previous studies are based on small series datasets, thus, they are less valuable for clinical guidance of SCA prognosis (4, 11, 18, 22–26, 28–32). Therefore, we carried out this large population-based research to have a better understanding of the clinical characteristics of SCA and established a convenient nomogram for clinical SCA prognosis prediction. We identified that primary site surgery, age, insurance, histologic type, tumor extension, WHO grade, chemotherapy, and PRT were independent prognostic factors for OS. While primary site surgery, insurance, tumor extension, PRT, histologic type, WHO grade, and chemotherapy were independent prognostic factors for CSS. Furthermore, the novel nomograms were confirmed superior to other single indicators for SCA 5- and 10-year survival prediction. In addition, we have developed web-based SCA online prognostic calculators for clinical utility.

Surgical procedures are preferred for patients with SCA to improve clinical symptoms and neurological function (11, 13, 14). In this study, 81.5% of the patients underwent surgical resection, while 20.3% for GTR, and 47.8% for partial resection. Surgery was found to be significantly associated with better survival in both OS and CSS. While the variable importance indicated the most correlated predictor for OS and CSS, respectively (Figures 4A,C). This is consistent with the previous studies (13, 18, 21, 24–26, 33–35). Adjuvant radiotherapy or chemotherapy using on SCA treatment is mainly based on the treatment protocol for brain gliomas and the efficacy still remains controversial (9, 18, 21, 29, 36). In this study, PRT and chemotherapy were associated with worse prognoses in both OS and CSS. The effect of PRT was consistent with many previous studies (21, 34, 35, 37) but inconsistencies had also emerged (18, 22, 24). Besides, few studies reported chemotherapy as the prognostic indicator (25, 29). We considered the poor prognosis of patients who received adjuvant therapy might be due to their tumor histologic type. Future studies should focus on the role of chemotherapy and radiotherapy for SCA prognosis, especially the difference between high-grade and low-grade SCA (24).

Histopathologically, SCA was divided into four subgroups according to the ICD-O-3 classification system in this study. Anaplastic astrocytoma, which belongs to high-grade SCA, accounted for 11.7% of the whole patient cohort. While 6.7 and 32.2% were for diffuse astrocytoma and pilocytic astrocytoma, respectively, which belong to low-grade SCA. This study revealed that anaplastic astrocytoma was significantly associated with a worse prognosis in both OS and CSS, compared to the other histologic type. These results coincided with many previous studies (26, 31, 35, 38), while others stated that histology made no difference in SCA survival (32, 39). This may be due to the selection bias of included patients, such as the sample size, tumor grade, histologic type, or surgical condition, etc. Moreover, histologic type demonstrated the obvious variable importance in prognosis prediction (Figures 4A,C). The WHO grade also remained the strong outcome predictor for OS and CSS in this study. As previous studies reported, it was considered a notable risk factor for SCA prognosis (18, 24, 25, 29, 34, 39).

Young age patients were found to have the better survival results for OS (*P* < 0.001) in this study. This finding was in line with the previous studies (18, 21, 22, 31, 35, 38). However, the HR value was close to one, indicating that the clinical values might not be so much significant. In addition, some published studies also stated that age was not related to SCA prognosis (24, 26, 29, 30, 32, 40). This might possibly be due to the small sample size. Nevertheless, age has shown excellent variable importance and predictive performance for OS prediction in this study. The multivariable Cox regressions also demonstrated that insurance and tumor extension were independent prognostic factors for OS and CSS. However, marital status was only discovered to be prognostic factors in univariable Cox regression but not multivariable regression for OS, while age and marital status for CSS. These findings were consistent with many previous studies (18, 25, 30, 32).

As a simple and convenient prediction tool, nomogram has its irreplaceable advantages in dealing with different variables that affect cancer patients' prognosis and has already been widely used in making the prediction of survival in malignant tumor patients (41–43). In this study, we established a convenient nomogram for intuitive clinical application in SCA prognosis prediction. The performance of the nomograms was evaluated by calibration curves, C-index, and ROC curves, while KM survival curves and DCA were used to evaluate the clinical utility. The nomograms demonstrated more accurate predictions for 5-/10-year OS and CSS of both training cohort and external validation cohort, compared to other single indicators. C-index for CSS prediction of patients with SCA was more than 0.8 while the 5-/10-year AUCs exceeded 0.85. These indicated that nomogram has the excellent performances in SCA survival prediction, which was similar to the study by Yuan et al. (25). We further developed risk discrimination systems that divided patients into high-risk, medium-risk, and low-risk groups according to the nomogram calculated total points. These might make a contribution to clinical patients' risk identification and treatments could be taken in time.

To our best knowledge, it's the largest sample size study of SCA prognosis, using the SEER database. Moreover, it's also the first to construct a web-based application for SCA survival prediction. The established nomogram had demonstrated good calibration, discrimination, and clinical utility, which might be a potential tool for other SCA outcome prediction such as recurrence, neurological outcome (e.g., paraplegia), adverse treatment effects (e.g., radiation toxicity), etc. However, there are still some limitations in this study. First, as a population-based study using the public SEER database, some important variables associated with the SCA prognosis are missing, such as neurological function, vertebral segments, molecular markers, imaging data, and genetic indicators. Second, as a rare tumor, there are still a limited amount of data used in this study though we have included the latest and most comprehensive data to collect as much data as possible. However, as the large time span of our data, advances in treatment modality over decades may lead to possible observation biases between patients. The treatment modality did show statistical differences between the decades (Supplementary Table 4). But the year of diagnosis was not found to be an independent factor for both OS and CSS in Cox regression analyses. Third, due to the limitations of the SEER database, some variables have missing values which may weaken the reliability. In order to reduce this impact and to maximize the statistical power, we only executed multiple imputations on variables with <70% missing values and excluded those with a larger degree of missing values (44, 45). To improve the robustness of imputation, 100 imputations were performed. Finally, based on data from the SEER registry, a population-based national database in the United States, the established nomograms and web-based applications were developed and validated. Thus, further validation using data from other countries would be helpful to improve the model's generalization ability. Despite these limitations, web-based online calculators are still practical and effective models for accurate and personalized prognostic prediction in patients with SCA.

## Conclusions

This study provides statistical evidence for understanding the clinical characteristics and risk predictors of SCA through a large population-based SEER registry. The established nomograms demonstrated good calibration, discrimination, and clinical utility. This might benefit clinical decision-making and patient management for SCA. In addition, the developed web-based online calculators would greatly improve clinical practicability and interpretability. More extensive external validation is needed before further use to improve the predictive applicability and generalization ability.

## Data Availability Statement

The raw data supporting the conclusions of this article will be made available by the authors, without undue reservation.

## Ethics Statement

Ethical review and approval was not required for the study on human participants in accordance with the local legislation and institutional requirements. Written informed consent from the participants' legal guardian/next of kin was not required to participate in this study in accordance with the national legislation and the institutional requirements.

## Author Contributions

SH, GF, and XL designed the study and critically revised the manuscript. SY was responsible for project implementation and administration. XY developed the prediction model and statistical analyses. HW extracted the data and performed data preprocessing. SY and YG wrote the original draft. JF and XQ performed the data visualization. CF conducted results interpretation. YL and LL edited the figures and tables. All authors reviewed and edited the final manuscript for submission.

## Funding

This work was funded by the National Natural Science Foundation of China (Grant no. 82102640) and Guangdong Basic and Applied Basic Research Foundation (Grant no. 2019A1515111171) granted to GF. The funders had no role in study design, data collection, data analysis, interpretation, writing of this report, and in the decision to submit the paper for publication.

## Conflict of Interest

The authors declare that the research was conducted in the absence of any commercial or financial relationships that could be construed as a potential conflict of interest.

## Publisher's Note

All claims expressed in this article are solely those of the authors and do not necessarily represent those of their affiliated organizations, or those of the publisher, the editors and the reviewers. Any product that may be evaluated in this article, or claim that may be made by its manufacturer, is not guaranteed or endorsed by the publisher.

## Abbreviations

PSCT: primary spinal cord tumor
CNS: central nervous systems
IMSCT: intramedullary spinal cord tumors
SCA: spinal cord astrocytoma
GTR: gross total resection
STR: subtotal resection
SEER: Surveillance, Epidemiology and End Results
ICD-O-3: International Classification of Disease for Oncology Version 3
PRT: postoperation radiotherapy
OS: overall survival
CSS: cancer-specific survival
HR: hazard ratio
CI: confidential interval
C-index: concordance index
ROC: receiver operating characteristic
DCA: decision curve analysis
KM: Kaplan-Meier
STROBE: Strengthening the Reporting of Observational Studies in Epidemiology
TRIPOD: Transparent Reporting of a multivariable prediction model for Individual Prognosis or Diagnosis
AUCs: area under the curves
AUDCs: area under the decision curves.
