# Supplementary material for: Development and Validation of a Personalized Prognostic Prediction Model for Patients With Spinal Cord Astrocytoma
Source: Front Med (Lausanne). 2022 Jan 18;8:802471. doi: 10.3389/fmed.2021.802471 (PMC8804494; doi:10.3389/fmed.2021.802471)
Supplement: Supplementary file 1 [file Data_Sheet_1.docx]

**Supplementary contents**

**Supplementary material A**

Supplementary table 1. Characteristics of patients before and after imputation in whole dataset.

Supplementary table 2. Missing values before imputation in whole dataset.

Supplementary table 3. Univariable analysis of overall survival (OS) and cancer-specific survival (CSS).

Supplementary table 4. Characteristics of patients between the decades.

Supplementary table 5. Score assignment for variables included in the nomograms of overall survival (OS) and cancer-specific survival (CSS).

Supplementary table 6. Evaluation of the nomogram prediction for overall survival (OS) compared to single indicators.

Supplementary table 7. Evaluation of the nomogram prediction for cancer-specific survival (CSS) compared to single indicators.

Supplementary figure 1. Kaplan-Meier survival curves of patients stratified by datasets for (A) Overall survival (OS). (B) Cancer-specific survival (CSS).

Supplementary figure 2. Evaluation of the nomogram on training dataset for cancer-specific survival (CSS). (A) 5- and 10-year Calibration plots of Nomogram. (B) 5-year and (C) 10-year Area Under the Curve (AUC) for Receiver Operating Characteristic (ROC) curves of Nomogram, Primary site surgery, Insurance and Tumor extension. (D) Overall Concordance Index (c-index) of Nomogram, Primary site surgery, Insurance and Tumor extension. (E) 5-year and 10-year Decision Curve Analysis (DCA) of Nomogram, Primary site surgery, Insurance and Tumor extension.

Supplementary figure 3. Evaluation of the nomogram on testing dataset for cancer-specific survival (CSS). (A) 5- and 10-year Calibration plots of Nomogram. (B) 5-year and (C) 10-year Area Under the Curve (AUC) for Receiver Operating Characteristic (ROC) curves of Nomogram, Primary site surgery, Insurance and Tumor extension. (D) Overall Concordance Index (c-index) of Nomogram, Primary site surgery, Insurance and Tumor extension. (E) 5-year and 10-year Decision Curve Analysis (DCA) of Nomogram, Primary site surgery, Insurance and Tumor extension.

Supplementary figure 4. Overall Area Under the Curve (AUC). (A) Training dataset of overall survival (OS). (B) Testing dataset of overall survival (OS). (C) Training dataset of cancer-specific survival (CSS). (D) Testing dataset of cancer-specific survival (CSS).

Supplementary figure 5. Cut-off values of nomogram total points calculated by X-tile. (A) Training dataset of Overall survival (OS). (B) Training dataset of cancer-specific survival (CSS).

Supplementary figure 6. The web survival rate calculator for (A) Overall survival (OS). (B) Cancer-specific survival (CSS).

Supplementary figure 7. The web survival rate calculator estimated overall survival (OS) of a hypothetical patient (60-year-old, insured, WHO grade III anaplastic astrocytoma with distant metastasis) based on different treatment strategy. (A) Survival curves of different treatments. (B) 5-year survival probability with 95%CI of different treatments. Abbreviations: GTR, gross total resection; PRT, postoperation radiotherapy; CT, chemotherapy.

Supplementary figure 8. The web survival rate calculator estimated cancer-specific survival (CSS) of a hypothetical patient (insured, WHO grade III anaplastic astrocytoma with distant metastasis) based on different treatment strategy. (A) Survival curves of different treatments. (B) 5-year survival probability with 95%CI of different treatments. Abbreviations: GTR, gross total resection; PRT, postoperation radiotherapy; CT, chemotherapy.

**Supplementary material B**

STROBE Statement for Checklist of items that should be included in reports of cohort studies

**Supplementary material C**

TRIPOD Checklist for Prediction Model Development and Validation

**Supplementary table 1. Characteristics of patients before and after imputation in whole dataset.**

| **Characteristics** | **Level** | **Before imputation (N= 818)** | **After imputation (N= 818)** | **P value** |
| --- | --- | --- | --- | --- |
| **OS (%)** | **Alive** | 478 (58.4) | 478 (58.4) | 1 |
|  | **Dead** | 340 (41.6) | 340 (41.6) |  |
| **CSS (%)** | **Alive** | 569 (69.6) | 569 (69.6) | 1 |
|  | **Dead** | 249 (30.4) | 249 (30.4) |  |
| **Survival.months (mean (SD))** | | 117.57 (113.51) | 117.57 (113.51) | 1 |
| **Year.of.diagnosis (%)** | **1970s** | 45 (5.5) | 45 (5.5) | 1 |
|  | **1980s** | 119 (14.5) | 119 (14.5) |  |
|  | **1990s** | 154 (18.8) | 154 (18.8) |  |
|  | **2000s** | 322 (39.4) | 322 (39.4) |  |
|  | **2010s** | 178 (21.8) | 178 (21.8) |  |
| **Age (mean (SD))** | | 30.84 (21.97) | 30.84 (21.97) | 1 |
| **Gender (%)** | **Female** | 340 (41.6) | 340 (41.6) | 1 |
|  | **Male** | 478 (58.4) | 478 (58.4) |  |
| **Race (%)** | **American Indian/Alaska Native** | 5 (0.6) | 5 (0.6) | 1 |
|  | **Asian or Pacific Islander** | 55 (6.8) | 55 (6.7) |  |
|  | **Black** | 110 (13.6) | 111 (13.6) |  |
|  | **White** | 640 (79.0) | 647 (79.1) |  |
| **Hispanic (%)** | **No** | 718 (87.8) | 718 (87.8) | 1 |
|  | **Yes** | 100 (12.2) | 100 (12.2) |  |
| **Insurance (%)** | **Insured** | 189 (73.8) | 492 (60.1) | <0.001 |
|  | **Uninsured/Medicaid** | 67 (26.2) | 326 (39.9) |  |
| **Marital.status (%)** | **Married** | 302 (37.9) | 314 (38.4) | 0.954 |
|  | **Separated/divorced/widowed** | 66 (8.3) | 70 (8.6) |  |
|  | **Single/unmarried** | 428 (53.8) | 434 (53.1) |  |
| **Residence (%)** | **Metropolitan** | 729 (90.6) | 740 (90.5) | 0.98 |
|  | **Rural/urban adjacent to metro area** | 45 (5.6) | 45 (5.5) |  |
|  | **Rural/urban not adjacent to metro area** | 31 (3.9) | 33 (4.0) |  |
| **At.least.bachelors.degree (%) (mean (SD))** | | 33.05 (10.80) | 33.05 (10.80) | 1 |
| **Families.below.poverty (%) (mean (SD))** | | 10.21 (4.45) | 10.21 (4.45) | 1 |
| **Unemployed (%) (mean (SD))** | | 6.94 (2.14) | 6.94 (2.14) | 1 |
| **Median.household income (in thousand) (mean (SD))** | | 65.91 (16.50) | 65.91 (16.50) | 1 |
| **Cost.of.living.index (in thousand) (mean (SD))** | | 1.03 (0.16) | 1.03 (0.16) | 1 |
| **Histologic.type (%)** | **Anaplastic astrocytoma** | 96 (11.7) | 96 (11.7) | 1 |
|  | **Astrocytoma, NOS** | 404 (49.4) | 404 (49.4) |  |
|  | **Diffuse astrocytoma** | 55 (6.7) | 55 (6.7) |  |
|  | **Pilocytic astrocytoma** | 263 (32.2) | 263 (32.2) |  |
| **WHO.grade (%)** | **I** | 270 (58.6) | 312 (38.1) | <0.001 |
|  | **II** | 92 (20.0) | 328 (40.1) |  |
|  | **III** | 99 (21.5) | 178 (21.8) |  |
| **Tumor.size (mm) (%)** | **＜28** | 96 (42.1) | 419 (51.2) | 0.018 |
|  | **≥28** | 132 (57.9) | 399 (48.8) |  |
| **Tumor.extension (%)** | **Distant** | 30 (4.0) | 31 (3.8) | 0.884 |
|  | **Localized** | 672 (89.7) | 731 (89.4) |  |
|  | **Regional** | 47 (6.3) | 56 (6.8) |  |
| **Primary.site.surgery (%)** | **Gross total resection** | 165 (20.4) | 166 (20.3) | 1 |
|  | **No surgery** | 148 (18.3) | 151 (18.5) |  |
|  | **Partial resection** | 386 (47.8) | 391 (47.8) |  |
|  | **Surgery, NOS** | 109 (13.5) | 110 (13.4) |  |
| **Postoperation.radiotherapy (%)** | **No** | 540 (66.3) | 540 (66.0) | 0.959 |
|  | **Yes** | 275 (33.7) | 278 (34.0) |  |
| **Chemotherapy (%)** | **No/Unknown** | 674 (82.4) | 674 (82.4) | 1 |
|  | **Yes** | 144 (17.6) | 144 (17.6) |  |

**Supplementary table 2. Missing values before imputation in whole dataset.**

| **Variables** | **Missing values** | **Total number** |
| --- | --- | --- |
| **OS** | 0 (0.00%) | 818 |
| **CSS** | 0 (0.00%) | 818 |
| **Survival.months** | 0 (0.00%) | 818 |
| **Year.of.diagnosis** | 0 (0.00%) | 818 |
| **Age** | 0 (0.00%) | 818 |
| **Gender** | 0 (0.00%) | 818 |
| **Race** | 8 (0.98%) | 818 |
| **Hispanic** | 0 (0.00%) | 818 |
| **Insurance** | 562 (68.7%) | 818 |
| **Marital.status** | 22 (2.69%) | 818 |
| **Residence** | 13 (1.59%) | 818 |
| **At.least.bachelors.degree** | 0 (0.00%) | 818 |
| **Families.below.poverty** | 0 (0.00%) | 818 |
| **Unemployed** | 0 (0.00%) | 818 |
| **Median.household.income.thousand.** | 0 (0.00%) | 818 |
| **Cost.of.living.index** | 0 (0.00%) | 818 |
| **Histologic.type** | 0 (0.00%) | 818 |
| **WHO.grade** | 357 (43.6%) | 818 |
| **Tumor.size** | 590 (72.1%) | 818 |
| **Tumor.extension** | 69 (8.44%) | 818 |
| **Primary.site.surgery** | 10 (1.22%) | 818 |
| **Postoperation.radiotherapy** | 3 (0.37%) | 818 |
| **Chemotherapy** | 0 (0.00%) | 818 |

**Supplementary table 3. Univariable analysis of overall survival (OS) and cancer-specific survival (CSS).**

| **Variables** | **Overall survival (OS)** | | **Cancer-specific survival (CSS)** | |
| --- | --- | --- | --- | --- |
|  | **HR (95%CI)** | **P value** | **HR (95%CI)** | **P value** |
| **Year.of.diagnosis** |  |  |  |  |
| **1980s VS 1970s** | 0.9 (0.56-1.45) | 0.659 | 0.83 (0.45-1.5) | 0.531 |
| **1990s VS 1970s** | 0.85 (0.52-1.37) | 0.5 | 0.82 (0.46-1.47) | 0.511 |
| **2000s VS 1970s** | 0.72 (0.45-1.16) | 0.181 | 0.7 (0.4-1.21) | 0.197 |
| **2010s VS 1970s** | 0.71 (0.4-1.25) | 0.236 | 0.67 (0.35-1.28) | 0.228 |
| **Age** | 1.03 (1.02-1.03) | <0.001 | 1.02 (1.01-1.02) | <0.001 |
| **Gender** |  |  |  |  |
| **Male VS Female** | 1.08 (0.83-1.4) | 0.556 | 1.22 (0.9-1.65) | 0.206 |
| **Race** |  |  |  |  |
| **Asian or Pacific Islander VS Indian/Alaska Native** | 0.57 (0.17-1.92) | 0.363 | 0.75 (0.17-3.25) | 0.706 |
| **Black VS Indian/Alaska Native** | 0.47 (0.14-1.51) | 0.202 | 0.52 (0.12-2.16) | 0.366 |
| **White VS Indian/Alaska Native** | 0.37 (0.12-1.16) | 0.089 | 0.44 (0.11-1.77) | 0.245 |
| **Hispanic** |  |  |  |  |
| **Yes VS No** | 0.97 (0.62-1.5) | 0.881 | 0.92 (0.56-1.52) | 0.745 |
| **Insurance** |  |  |  |  |
| **Uninsured/Medicaid VS Insured** | 0.46 (0.35-0.61) | <0.001 | 0.35 (0.24-0.49) | <0.001 |
| **Marital.status** | |  |  |  |
| **Separated/divorced/widowed VS Married** | 1.27 (0.82-1.97) | 0.292 | 1.15 (0.67-1.97) | 0.611 |
| **Single/unmarried VS Married** | 0.57 (0.43-0.74) | <0.001 | 0.73 (0.54-1) | 0.048 |
| **Residence** |  |  |  |  |
| **Rural/urban adjacent to metro area VS Metropolitan** | 1.55 (0.92-2.63) | 0.099 | 1.64 (0.91-2.95) | 0.1 |
| **Rural/urban not adjacent to metro area VS Metropolitan** | 1.35 (0.73-2.47) | 0.336 | 1.2 (0.59-2.44) | 0.615 |
| **At.least.bachelors.degree** | 1 (0.99-1.01) | 0.703 | 0.99 (0.98-1.01) | 0.212 |
| **Families.below.poverty** | 1.02 (0.99-1.05) | 0.282 | 1.02 (0.99-1.05) | 0.239 |
| **Unemployed** | 1 (0.94-1.06) | 0.919 | 0.98 (0.91-1.05) | 0.558 |
| **Median.household.income.thousand.** | 1 (0.99-1) | 0.43 | 0.99 (0.99-1) | 0.258 |
| **Cost.of.living.index** | 1.22 (0.53-2.82) | 0.644 | 0.98 (0.37-2.59) | 0.972 |
| **Histologic.type** | |  |  |  |
| **Astrocytoma, NOS VS Anaplastic astrocytoma** | 0.38 (0.27-0.54) | <0.001 | 0.31 (0.22-0.45) | <0.001 |
| **Diffuse astrocytoma VS Anaplastic astrocytoma** | 0.52 (0.3-0.9) | 0.021 | 0.39 (0.21-0.73) | 0.003 |
| **Pilocytic astrocytoma VS Anaplastic astrocytoma** | 0.14 (0.09-0.22) | <0.001 | 0.09 (0.05-0.16) | <0.001 |
| **WHO.grade** | |  |  |  |
| **II VS I** | 3.94 (2.68-5.79) | <0.001 | 4.36 (2.7-7.02) | <0.001 |
| **III VS I** | 4.84 (3.22-7.29) | <0.001 | 7.3 (4.47-11.92) | <0.001 |
| **Tumor.size** | |  |  |  |
| **≥28 VS ＜28** | 0.81 (0.62-1.04) | 0.103 | 0.88 (0.65-1.18) | 0.381 |
| **Tumor.extension** | |  |  |  |
| **Localized VS Distant** | 0.5 (0.29-0.88) | 0.016 | 0.38 (0.22-0.67) | 0.001 |
| **Regional VS Distant** | 0.9 (0.46-1.74) | 0.752 | 0.83 (0.42-1.64) | 0.587 |
| **Primary.site.surgery** | |  |  |  |
| **No surgery VS Gross total resection** | 4.96 (3.14-7.83) | <0.001 | 6.3 (3.56-11.16) | <0.001 |
| **Partial resection VS Gross total resection** | 2.32 (1.49-3.62) | <0.001 | 2.88 (1.65-5.02) | <0.001 |
| **Surgery, NOS VS Gross total resection** | 2.53 (1.55-4.12) | <0.001 | 3.23 (1.74-5.99) | <0.001 |
| **Postoperation.radiotherapy** | | |  |  |
| **Yes VS No** | 1.84 (1.42-2.38) | <0.001 | 2.06 (1.53-2.77) | <0.001 |
| **Chemotherapy** | |  |  |  |
| **Yes VS No/Unknown** | 2.45 (1.81-3.32) | <0.001 | 2.7 (1.94-3.74) | <0.001 |

**Supplementary table 4. Characteristics of patients between the decades.**

| **Characteristics** | **level** | **Overall (N=818)** | **1970s (N=45)** | **1980s (N=119)** | **1990s (N=154)** | **2000s (N=322)** | **2010s (N=178)** | **P value** |
| --- | --- | --- | --- | --- | --- | --- | --- | --- |
| **OS (%)** | **Alive** | 478 (58.4) | 11 (24.4) | 45 (37.8) | 73 (47.4) | 209 (64.9) | 140 (78.7) | <0.001 |
|  | **Dead** | 340 (41.6) | 34 (75.6) | 74 (62.2) | 81 (52.6) | 113 (35.1) | 38 (21.3) |  |
| **CSS (%)** | **Alive** | 569 (69.6) | 25 (55.6) | 71 (59.7) | 98 (63.6) | 231 (71.7) | 144 (80.9) | <0.001 |
|  | **Dead** | 249 (30.4) | 20 (44.4) | 48 (40.3) | 56 (36.4) | 91 (28.3) | 34 (19.1) |  |
| **Survival.months (mean (SD))** | | 117.57 (113.51) | 212.80 (193.96) | 190.82 (158.45) | 156.58 (114.57) | 103.48 (62.52) | 36.24 (26.51) | <0.001 |
| **Age (mean (SD))** | | 30.84 (21.97) | 28.13 (19.31) | 30.24 (19.77) | 31.85 (21.68) | 28.80 (22.38) | 34.73 (23.12) | 0.05 |
| **Gender (%)** | **Female** | 340 (41.6) | 21 (46.7) | 54 (45.4) | 58 (37.7) | 132 (41.0) | 75 (42.1) | 0.694 |
|  | **Male** | 478 (58.4) | 24 (53.3) | 65 (54.6) | 96 (62.3) | 190 (59.0) | 103 (57.9) |  |
| **Race (%)** | **American Indian/Alaska Native** | 5 (0.6) | 0 (0.0) | 1 (0.8) | 2 (1.3) | 1 (0.3) | 1 (0.6) | 0.463 |
|  | **Asian or Pacific Islander** | 55 (6.7) | 3 (6.7) | 4 (3.4) | 10 (6.5) | 26 (8.1) | 12 (6.7) |  |
|  | **Black** | 111 (13.6) | 2 (4.4) | 18 (15.1) | 28 (18.2) | 40 (12.4) | 23 (12.9) |  |
|  | **White** | 647 (79.1) | 40 (88.9) | 96 (80.7) | 114 (74.0) | 255 (79.2) | 142 (79.8) |  |
| **Hispanic (%)** | **No** | 718 (87.8) | 43 (95.6) | 115 (96.6) | 144 (93.5) | 263 (81.7) | 153 (86.0) | <0.001 |
|  | **Yes** | 100 (12.2) | 2 (4.4) | 4 (3.4) | 10 (6.5) | 59 (18.3) | 25 (14.0) |  |
| **Insurance (%)** | **Insured** | 492 (60.1) | 24 (53.3) | 53 (44.5) | 80 (51.9) | 195 (60.6) | 140 (78.7) | <0.001 |
|  | **Uninsured/Medicaid** | 326 (39.9) | 21 (46.7) | 66 (55.5) | 74 (48.1) | 127 (39.4) | 38 (21.3) |  |
| **Marital.status (%)** | **Married** | 314 (38.4) | 19 (42.2) | 45 (37.8) | 64 (41.6) | 112 (34.8) | 74 (41.6) | 0.638 |
|  | **Separated/divorced/widowed** | 70 (8.6) | 2 (4.4) | 14 (11.8) | 13 (8.4) | 28 (8.7) | 13 (7.3) |  |
|  | **Single/unmarried** | 434 (53.1) | 24 (53.3) | 60 (50.4) | 77 (50.0) | 182 (56.5) | 91 (51.1) |  |
| **Residence (%)** | **Metropolitan** | 740 (90.5) | 42 (93.3) | 104 (87.4) | 147 (95.5) | 292 (90.7) | 155 (87.1) | 0.185 |
|  | **Rural/urban adjacent to metro area** | 45 (5.5) | 1 (2.2) | 11 (9.2) | 4 (2.6) | 16 (5.0) | 13 (7.3) |  |
|  | **Rural/urban not adjacent to metro area** | 33 (4.0) | 2 (4.4) | 4 (3.4) | 3 (1.9) | 14 (4.3) | 10 (5.6) |  |
| **At.least.bachelors.degree (%) (mean (SD))** | | 33.05 (10.80) | 37.39 (10.75) | 33.84 (11.53) | 35.51 (10.15) | 31.78 (10.31) | 31.61 (11.14) | <0.001 |
| **Families.below.poverty (%) (mean (SD))** | | 10.21 (4.45) | 8.87 (4.24) | 9.41 (4.25) | 9.99 (4.15) | 10.64 (4.59) | 10.52 (4.54) | 0.016 |
| **Unemployed (%) (mean (SD))** | | 6.94 (2.14) | 6.52 (2.34) | 6.48 (2.42) | 6.88 (2.24) | 7.06 (1.97) | 7.19 (2.07) | 0.029 |
| **Median.household.income (in thousand) (mean (SD))** | | 65.91 (16.50) | 70.62 (16.77) | 65.26 (16.79) | 67.07 (15.62) | 65.41 (16.50) | 65.05 (16.92) | 0.25 |
| **Cost.of.living.index (in thousand) (mean (SD))** | | 1.03 (0.16) | 1.07 (0.15) | 1.02 (0.17) | 1.06 (0.13) | 1.03 (0.16) | 1.02 (0.17) | 0.116 |
| **Histologic.type (%)** | **Anaplastic astrocytoma** | 96 (11.7) | 2 (4.4) | 6 (5.0) | 30 (19.5) | 35 (10.9) | 23 (12.9) | <0.001 |
|  | **Astrocytoma, NOS** | 404 (49.4) | 34 (75.6) | 98 (82.4) | 72 (46.8) | 140 (43.5) | 60 (33.7) |  |
|  | **Diffuse astrocytoma** | 55 (6.7) | 7 (15.6) | 6 (5.0) | 14 (9.1) | 18 (5.6) | 10 (5.6) |  |
|  | **Pilocytic astrocytoma** | 263 (32.2) | 2 (4.4) | 9 (7.6) | 38 (24.7) | 129 (40.1) | 85 (47.8) |  |
| **WHO.grade (%)** | **I** | 312 (38.1) | 2 (4.4) | 17 (14.3) | 51 (33.1) | 151 (46.9) | 91 (51.1) | <0.001 |
|  | **II** | 328 (40.1) | 27 (60.0) | 70 (58.8) | 53 (34.4) | 118 (36.6) | 60 (33.7) |  |
|  | **III** | 178 (21.8) | 16 (35.6) | 32 (26.9) | 50 (32.5) | 53 (16.5) | 27 (15.2) |  |
| **Tumor.size (mm) (%)** | **＜28** | 419 (51.2) | 29 (64.4) | 79 (66.4) | 73 (47.4) | 150 (46.6) | 88 (49.4) | 0.001 |
|  | **≥28** | 399 (48.8) | 16 (35.6) | 40 (33.6) | 81 (52.6) | 172 (53.4) | 90 (50.6) |  |
| **Tumor.extension (%)** | **Distant** | 31 (3.8) | 0 (0.0) | 3 (2.5) | 6 (3.9) | 18 (5.6) | 4 (2.2) | 0.229 |
|  | **Localized** | 731 (89.4) | 42 (93.3) | 108 (90.8) | 132 (85.7) | 284 (88.2) | 165 (92.7) |  |
|  | **Regional** | 56 (6.8) | 3 (6.7) | 8 (6.7) | 16 (10.4) | 20 (6.2) | 9 (5.1) |  |
| **Primary.site.surgery (%)** | **Gross total resection** | 166 (20.3) | 0 (0.0) | 11 (9.2) | 56 (36.4) | 88 (27.3) | 11 (6.2) | <0.001 |
|  | **No surgery** | 151 (18.5) | 15 (33.3) | 26 (21.8) | 31 (20.1) | 58 (18.0) | 21 (11.8) |  |
|  | **Partial resection** | 391 (47.8) | 1 (2.2) | 10 (8.4) | 65 (42.2) | 171 (53.1) | 144 (80.9) |  |
|  | **Surgery, NOS** | 110 (13.4) | 29 (64.4) | 72 (60.5) | 2 (1.3) | 5 (1.6) | 2 (1.1) |  |
| **Postoperation.radiotherapy (%)** | **No** | 540 (66.0) | 27 (60.0) | 69 (58.0) | 99 (64.3) | 239 (74.2) | 106 (59.6) | 0.002 |
|  | **Yes** | 278 (34.0) | 18 (40.0) | 50 (42.0) | 55 (35.7) | 83 (25.8) | 72 (40.4) |  |
| **Chemotherapy (%)** | **No/Unknown** | 674 (82.4) | 43 (95.6) | 110 (92.4) | 135 (87.7) | 257 (79.8) | 129 (72.5) | <0.001 |
|  | **Yes** | 144 (17.6) | 2 (4.4) | 9 (7.6) | 19 (12.3) | 65 (20.2) | 49 (27.5) |  |

**Supplementary table 5.** **Score assignment for variables included in the nomograms of overall survival (OS) and cancer-specific survival (CSS).**

| **Overall survival (OS)** | | | **Cancer-specific survival (CSS)** | | |
| --- | --- | --- | --- | --- | --- |
| **Variables** | **Category** | **Score** | **Variables** | **Category** | **Score** |
| **Age** | 0 | 0 | **Insurance** | Insured | 55 |
|  | 10 | 10 |  | Uninsured/Medicaid | 0 |
|  | 20 | 20 | **Histologic.type** | Anaplastic astrocytoma | 40 |
|  | 30 | 30 |  | Astrocytoma, NOS | 0 |
|  | 40 | 40 |  | Diffuse astrocytoma | 8 |
|  | 50 | 50 |  | Pilocytic astrocytoma | 31 |
|  | 60 | 60 | **WHO.grade** | I | 0 |
|  | 70 | 70 |  | II | 78 |
|  | 80 | 80 |  | III | 80 |
|  | 90 | 90 | **Tumor.extension** | Distant | 48 |
| **Insurance** | Insured | 41 |  | Localized | 0 |
|  | Uninsured/  Medicaid | 0 |  | Regional | 26 |
| **Histologic.type** | Anaplastic astrocytoma | 65 | **Primary.site.**  **surgery** | Gross total resection | 0 |
|  | Astrocytoma, NOS | 0 |  | No surgery | 100 |
|  | Diffuse astrocytoma | 16 |  | Partial resection | 40 |
|  | Pilocytic astrocytoma | 55 |  | Surgery, NOS | 51 |
| **WHO.grade** | I | 0 | **Postoperation.**  **radiotherapy** | No | 0 |
|  | II | 100 |  | Yes | 35 |
|  | III | 76 | **Chemotherapy** | No/Unknown | 0 |
| **Tumor.extension** | Distant | 47 |  | Yes | 17 |
|  | Localized | 0 |  |  |  |
|  | Regional | 28 |  |  |  |
| **Primary.site.**  **surgery** | Gross total resection | 0 |  |  |  |
|  | No surgery | 100 |  |  |  |
|  | Partial resection | 36 |  |  |  |
|  | Surgery, NOS | 53 |  |  |  |
| **Postoperation.**  **radiotherapy** | No | 0 |  |  |  |
|  | Yes | 29 |  |  |  |
| **Chemotherapy** | No/Unknown | 0 |  |  |  |
|  | Yes | 30 |  | |  |
| **5-year Survival** | |  | **5-year Survival** | |  |
| **Total scores** | **5-year Survival Probability** | | **Total scores** | **5-year Survival Probability** | |
| 335 | 0.1 |  | 278 | 0.1 |  |
| 314 | 0.2 |  | 261 | 0.2 |  |
| 297 | 0.3 |  | 247 | 0.3 |  |
| 280 | 0.4 |  | 234 | 0.4 |  |
| 264 | 0.5 |  | 221 | 0.5 |  |
| 246 | 0.6 |  | 206 | 0.6 |  |
| 224 | 0.7 |  | 189 | 0.7 |  |
| 196 | 0.8 |  | 167 | 0.8 |  |
| 152 | 0.9 |  | 131 | 0.9 |  |
| **10-year Survival** | |  | **10-year Survival** | |  |
| **Total scores** | **10-year Survival Probability** | | **Total scores** | **10-year Survival Probability** | |
| 311 | 0.1 |  | 263 | 0.1 |  |
| 290 | 0.2 |  | 246 | 0.2 |  |
| 273 | 0.3 |  | 233 | 0.3 |  |
| 256 | 0.4 |  | 220 | 0.4 |  |
| 240 | 0.5 |  | 206 | 0.5 |  |
| 222 | 0.6 |  | 192 | 0.6 |  |
| 200 | 0.7 |  | 175 | 0.7 |  |
| 172 | 0.8 |  | 152 | 0.8 |  |
| 128 | 0.9 |  | 117 | 0.9 |  |

**Supplementary table 6.** **Evaluation of the nomogram prediction for overall survival (OS) compared to single indicators.**

| **Dataset** | **Model** | **Mean c-index** | **5-year AUC** | **10-year AUC** | **mean AUC** | **5-year AUDC** | **10-year AUDC** |
| --- | --- | --- | --- | --- | --- | --- | --- |
| **Training dataset** | **Nomogram** | 0.783 | 0.82 | 0.849 | 0.833 | 0.087 | 0.155 |
|  | **Primary site surgery** | 0.631 | 0.545 | 0.536 | 0.543 | 0.024 | 0.038 |
|  | **Age** | 0.619 | 0.613 | 0.67 | 0.66 | 0.02 | 0.047 |
|  | **Insurance** | 0.609 | 0.509 | 0.507 | 0.509 | 0.012 | 0.018 |
|  | **Histologic type** | 0.656 | 0.515 | 0.512 | 0.514 | 0.037 | 0.048 |
|  | **Tumor extension** | 0.539 | 0.524 | 0.519 | 0.523 | 0.003 | 0.004 |
|  | **WHO grade** | 0.66 | 0.693 | 0.652 | 0.635 | 0.027 | 0.038 |
|  | **Chemotherapy** | 0.565 | 0.512 | 0.51 | 0.511 | 0.008 | 0.01 |
|  | **Postoperation radiotherapy** | 0.576 | 0.503 | 0.502 | 0.503 | 0.006 | 0.009 |
| **Testing dataset** | **Nomogram** | 0.769 | 0.843 | 0.881 | 0.855 | 0.114 | 0.184 |
|  | **Primary site surgery** | 0.566 | 0.58 | 0.567 | 0.578 | 0.028 | 0.037 |
|  | **Age** | 0.667 | 0.701 | 0.761 | 0.754 | 0.039 | 0.067 |
|  | **Insurance** | 0.601 | 0.512 | 0.51 | 0.512 | 0.019 | 0.031 |
|  | **Histologic type** | 0.695 | 0.562 | 0.551 | 0.56 | 0.062 | 0.08 |
|  | **Tumor extension** | 0.526 | 0.624 | 0.603 | 0.621 | 0.007 | 0.007 |
|  | **WHO grade** | 0.673 | 0.732 | 0.712 | 0.666 | 0.043 | 0.054 |
|  | **Chemotherapy** | 0.551 | 0.519 | 0.515 | 0.518 | 0.015 | 0.016 |
|  | **Postoperation radiotherapy** | 0.6 | 0.506 | 0.505 | 0.506 | 0.015 | 0.018 |

**Supplementary table 7. Evaluation of the nomogram prediction for cancer-specific survival (CSS) compared to single indicators.**

| **Dataset** | **Model** | **Mean c-index** | **5-year AUC** | **10-year AUC** | **mean AUC** | **5-year AUDC** | **10-year AUDC** |
| --- | --- | --- | --- | --- | --- | --- | --- |
| **Training dataset** | **Nomogram** | 0.806 | 0.851 | 0.87 | 0.855 | 0.089 | 0.136 |
|  | **Primary site surgery** | 0.639 | 0.54 | 0.533 | 0.539 | 0.025 | 0.037 |
|  | **Insurance** | 0.621 | 0.507 | 0.505 | 0.507 | 0.016 | 0.022 |
|  | **Tumor extension** | 0.55 | 0.52 | 0.516 | 0.52 | 0.004 | 0.006 |
|  | **Postoperation radiotherapy** | 0.585 | 0.503 | 0.503 | 0.503 | 0.007 | 0.01 |
|  | **Histologic type** | 0.672 | 0.513 | 0.511 | 0.513 | 0.038 | 0.048 |
|  | **WHO grade** | 0.683 | 0.712 | 0.674 | 0.659 | 0.031 | 0.04 |
|  | **Chemotherapy** | 0.577 | 0.51 | 0.508 | 0.51 | 0.008 | 0.011 |
| **Testing dataset** | **Nomogram** | 0.762 | 0.834 | 0.858 | 0.835 | 0.084 | 0.124 |
|  | **Primary site surgery** | 0.551 | 0.572 | 0.563 | 0.575 | 0.025 | 0.027 |
|  | **Insurance** | 0.607 | 0.514 | 0.513 | 0.515 | 0.019 | 0.025 |
|  | **Tumor extension** | 0.52 | 0.609 | 0.595 | 0.613 | 0.006 | 0.005 |
|  | **Postoperation radiotherapy** | 0.603 | 0.507 | 0.506 | 0.507 | 0.012 | 0.012 |
|  | **Histologic type** | 0.702 | 0.565 | 0.557 | 0.567 | 0.053 | 0.071 |
|  | **WHO grade** | 0.692 | 0.738 | 0.726 | 0.683 | 0.038 | 0.045 |
|  | **Chemotherapy** | 0.564 | 0.522 | 0.519 | 0.522 | 0.011 | 0.011 |


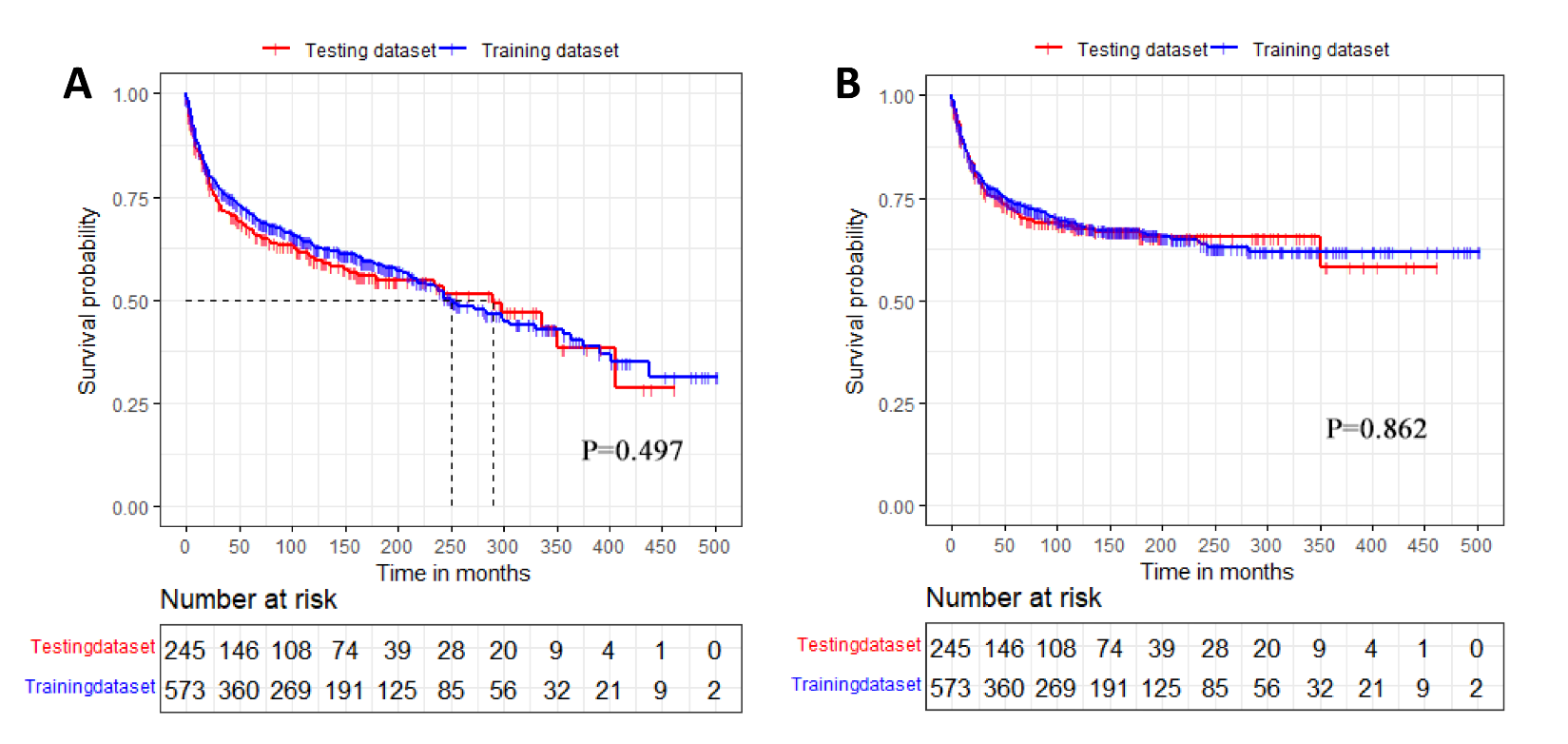


Supplementary figure 1. Kaplan-Meier survival curves of patients stratified by datasets for (A) Overall survival (OS). (B) Cancer-specific survival (CSS).


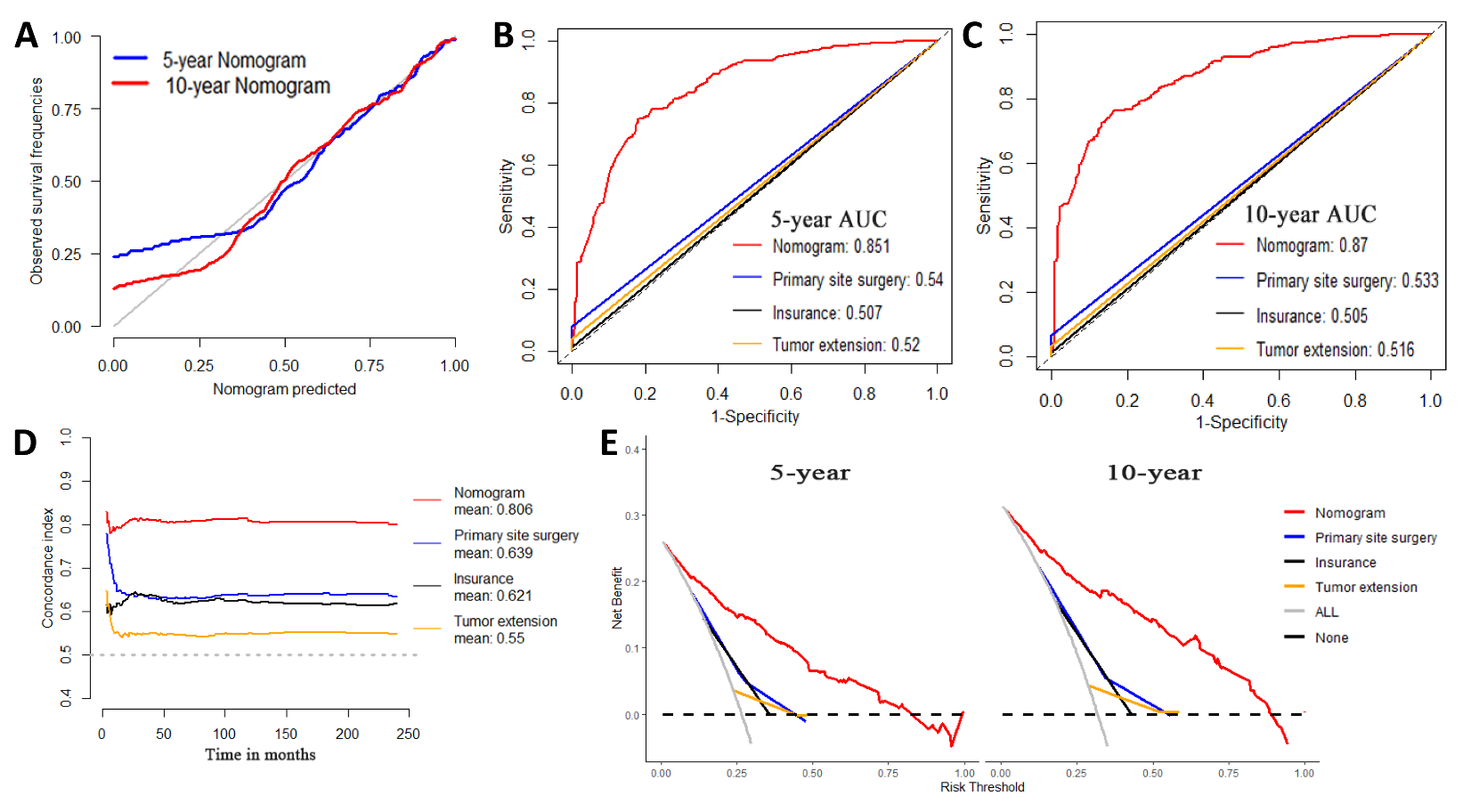


Supplementary figure 2. Evaluation of the nomogram on training dataset for cancer-specific survival (CSS). (A) 5- and 10-year Calibration plots of Nomogram. (B) 5-year and (C) 10-year Area Under the Curve (AUC) for Receiver Operating Characteristic (ROC) curves of Nomogram, Primary site surgery, Insurance and Tumor extension. (D) Overall Concordance Index (c-index) of Nomogram, Primary site surgery, Insurance and Tumor extension. (E) 5-year and 10-year Decision Curve Analysis (DCA) of Nomogram, Primary site surgery, Insurance and Tumor extension.


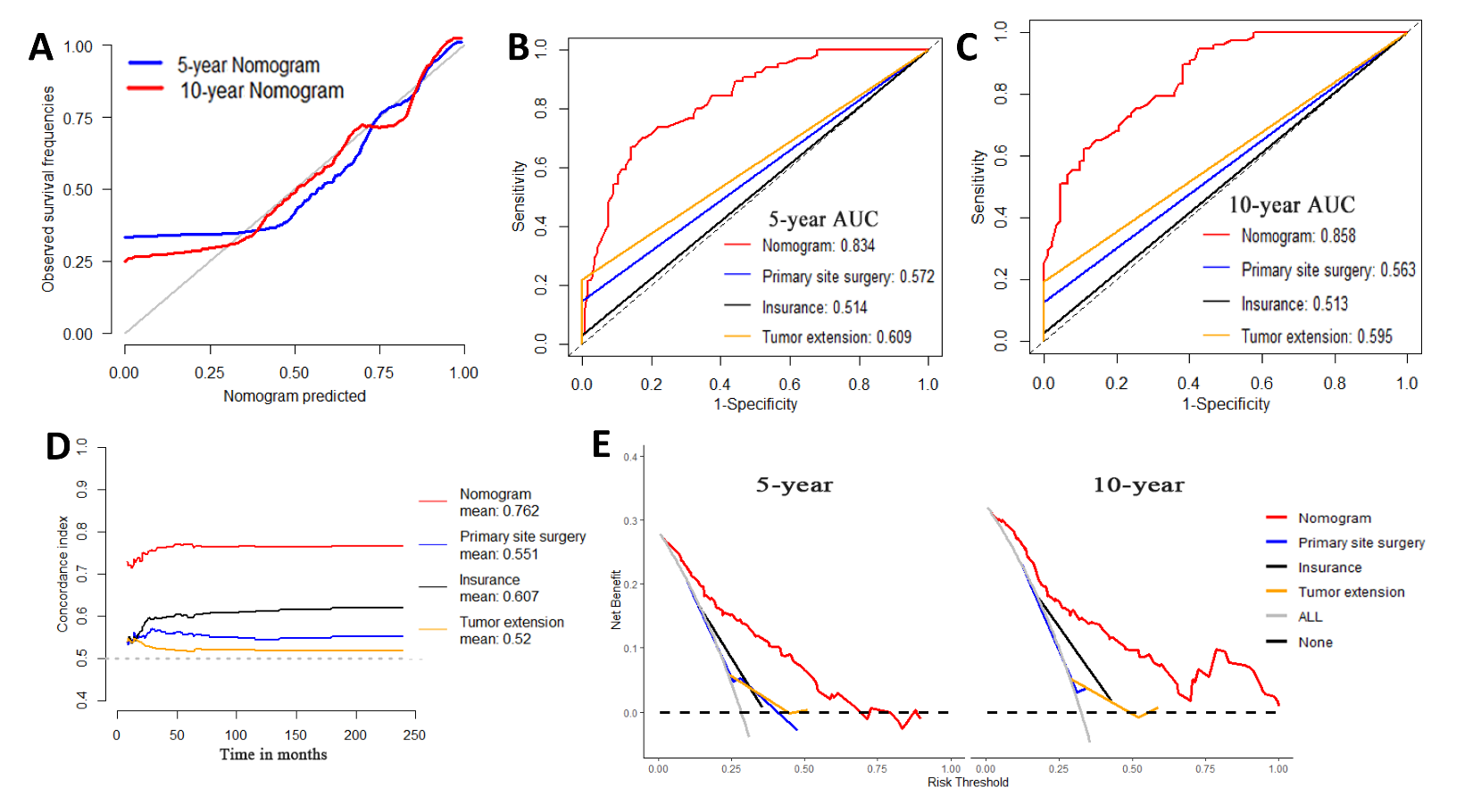


Supplementary figure 3. Evaluation of the nomogram on testing dataset for cancer-specific survival (CSS). (A) 5- and 10-year Calibration plots of Nomogram. (B) 5-year and (C) 10-year Area Under the Curve (AUC) for Receiver Operating Characteristic (ROC) curves of Nomogram, Primary site surgery, Insurance and Tumor extension. (D) Overall Concordance Index (c-index) of Nomogram, Primary site surgery, Insurance and Tumor extension. (E) 5-year and 10-year Decision Curve Analysis (DCA) of Nomogram, Primary site surgery, Insurance and Tumor extension.


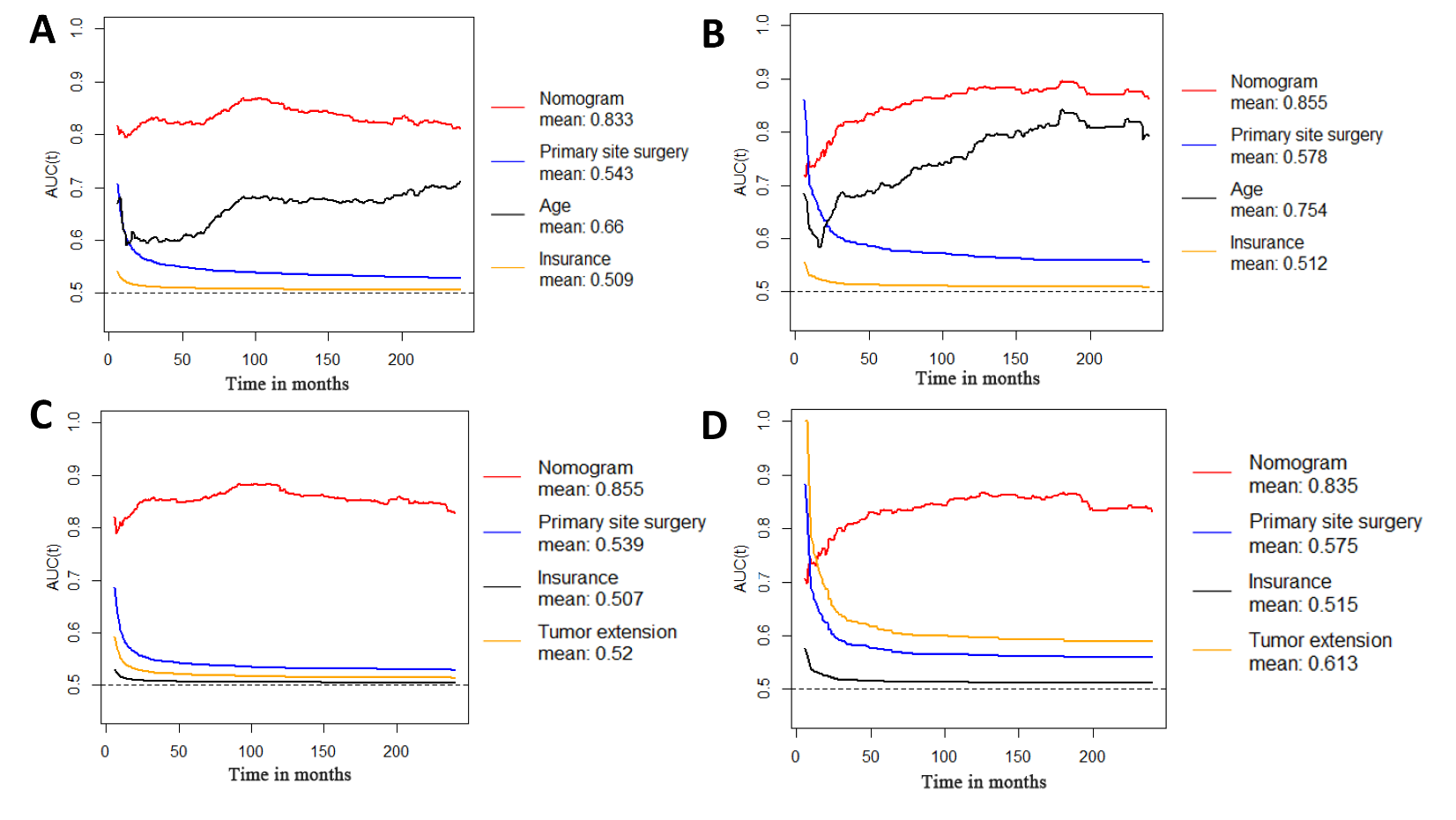


Supplementary figure 4. Overall Area Under the Curve (AUC). (A) Training dataset of overall survival (OS). (B) Testing dataset of overall survival (OS). (C) Training dataset of cancer-specific survival (CSS). (D) Testing dataset of cancer-specific survival (CSS).


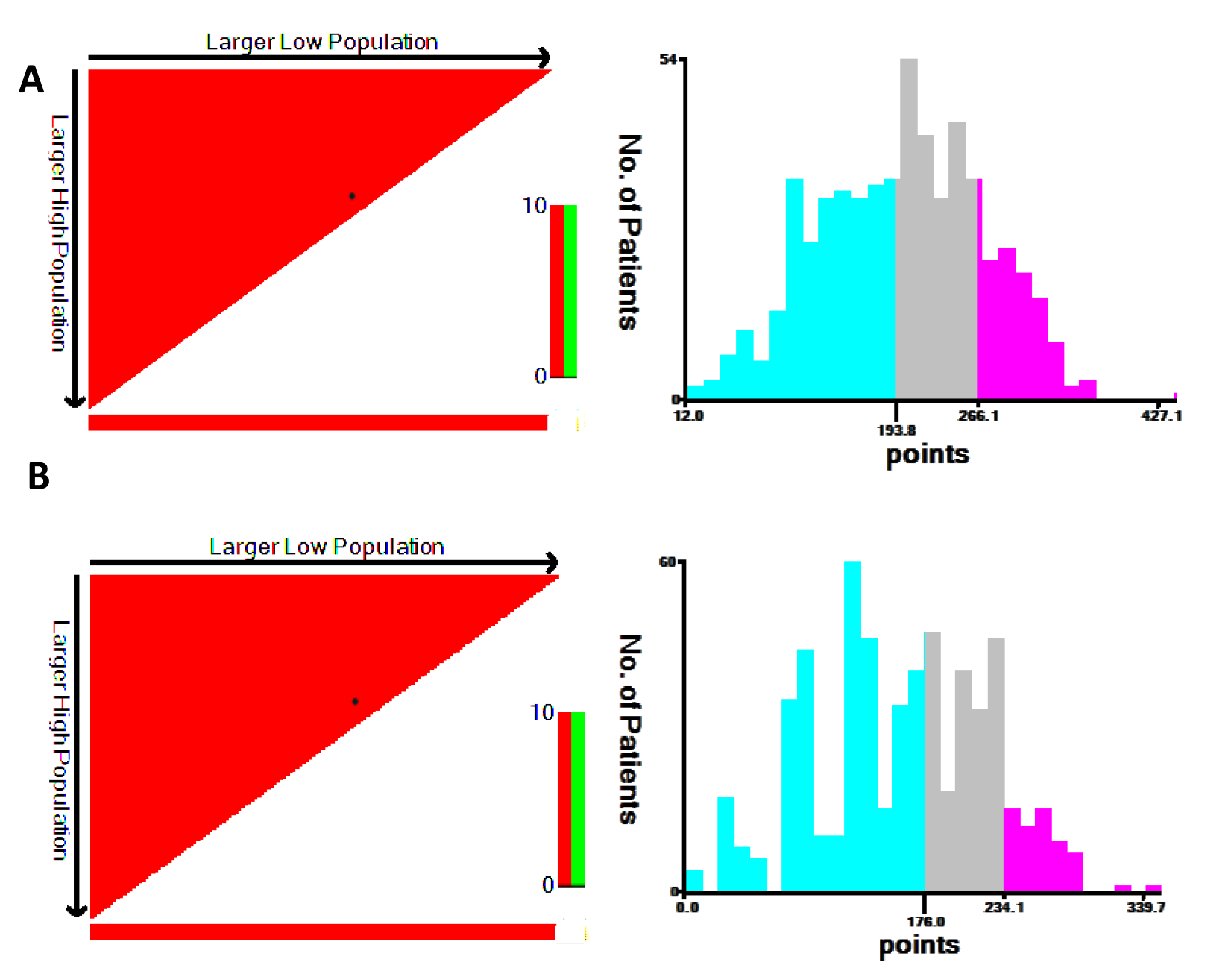


Supplementary figure 5. Cut-off values of nomogram total points calculated by X-tile. (A) Training dataset of Overall survival (OS). (B) Training dataset of cancer-specific survival (CSS).


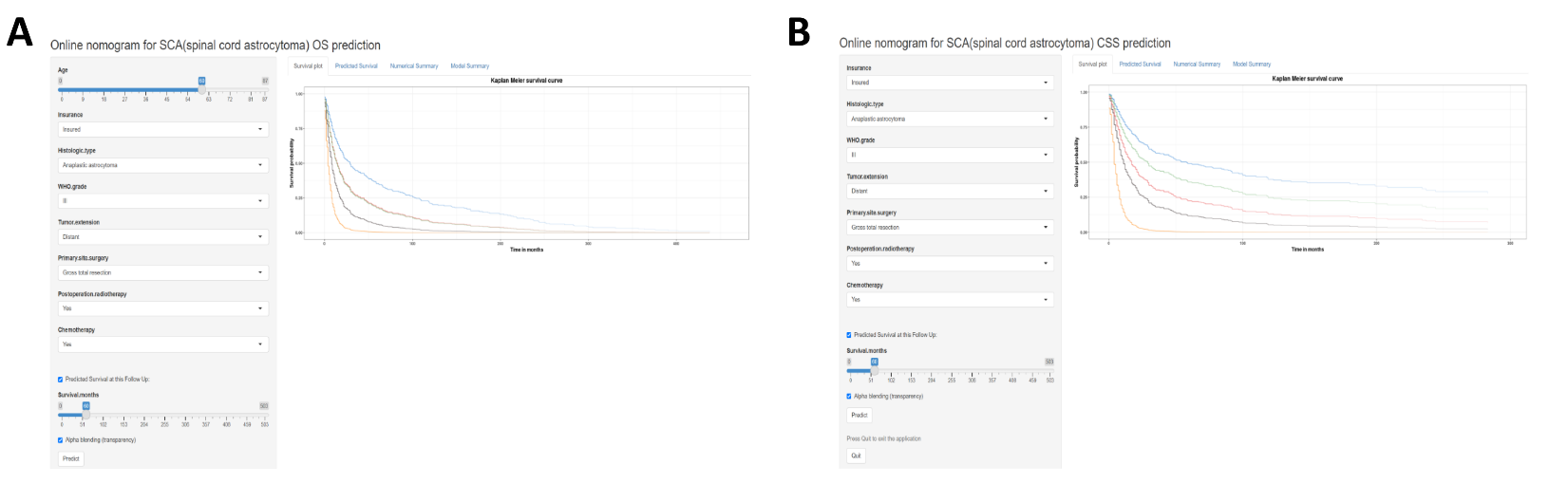


Supplementary figure 6. The web survival rate calculator for (A) Overall survival (OS). (B) Cancer-specific survival (CSS).


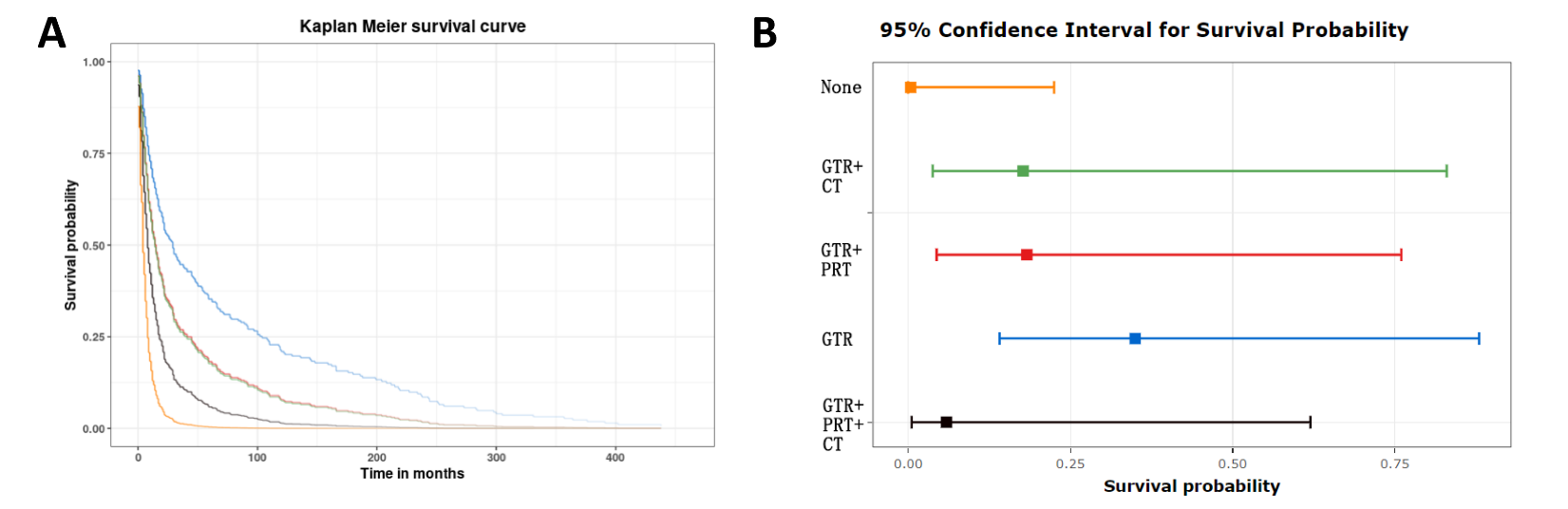


Supplementary figure 7. The web survival rate calculator estimated overall survival (OS) of a hypothetical patient (60-year-old, insured, WHO grade III anaplastic astrocytoma with distant metastasis) based on different treatment strategy. (A) Survival curves of different treatments. (B) 5-year survival probability with 95%CI of different treatments. Abbreviations: GTR, gross total resection; PRT, postoperation radiotherapy; CT, chemotherapy.


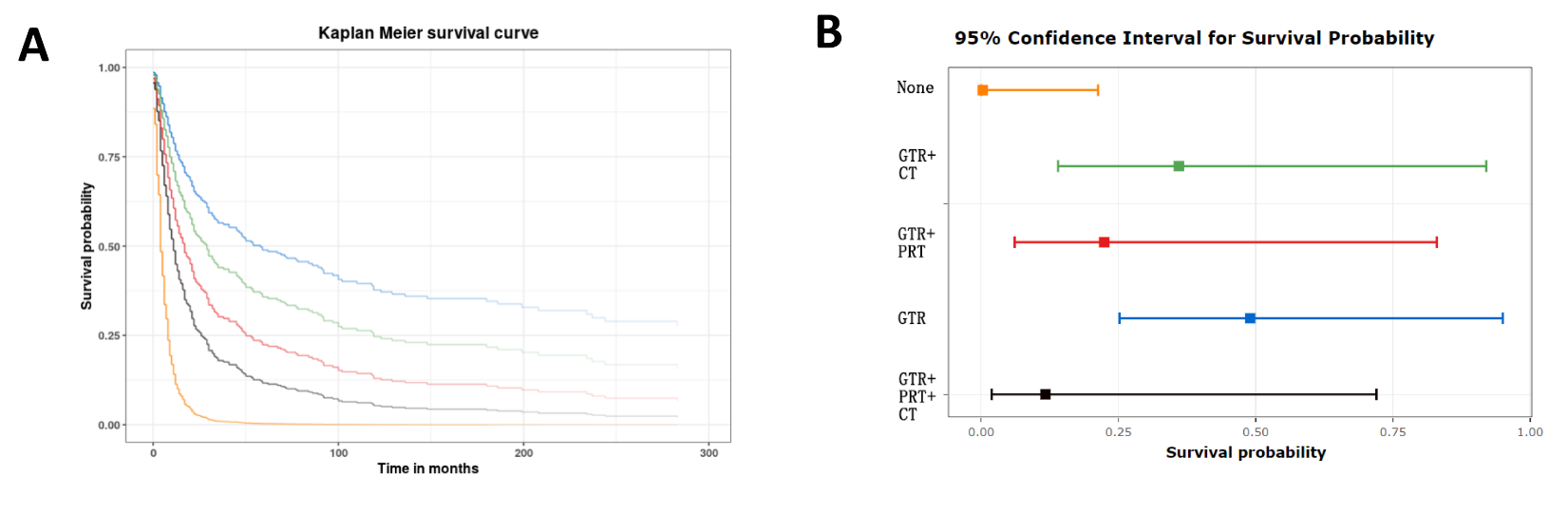


Supplementary figure 8. The web survival rate calculator estimated cancer-specific survival (CSS) of a hypothetical patient (insured, WHO grade III anaplastic astrocytoma with distant metastasis) based on different treatment strategy. (A) Survival curves of different treatments. (B) 5-year survival probability with 95%CI of different treatments. Abbreviations: GTR, gross total resection; PRT, postoperation radiotherapy; CT, chemotherapy.
